# XRCC1: a potential prognostic and immunological biomarker in LGG based on systematic pan-cancer analysis

**DOI:** 10.18632/aging.205426

**Published:** 2024-01-12

**Authors:** Guobing Wang, Yunyue Li, Rui Pan, Xisheng Yin, Congchao Jia, Yuchen She, Luling Huang, Guanhu Yang, Hao Chi, Gang Tian

**Affiliations:** 1 Department of Laboratory Medicine, The Affiliated Hospital of Southwest Medical University, Luzhou, China; 2 Medical Clinical Laboratory, Yibin Hospital of T.C.M, Yibin, China; 3 Queen Mary College, Medical School of Nanchang University, Nanchang, China; 4 Clinical Medical College, Southwest Medical University, Luzhou, China; 5 Department of Dermatology, Xijing Hospital, Fourth Military Medical University, Xi'an, China; 6 Department of Specialty Medicine, Ohio University, Athens, OH 45701, USA

**Keywords:** X-ray repair cross-complementation group 1, pan-cancer, prognosis, immune infiltration, tumor microenvironment

## Abstract

X-ray repair cross-complementation group 1 (XRCC1) is a pivotal contributor to base excision repair, and its dysregulation has been implicated in the oncogenicity of various human malignancies. However, a comprehensive pan-cancer analysis investigating the prognostic value, immunological functions, and epigenetic associations of XRCC1 remains lacking. To address this knowledge gap, we conducted a systematic investigation employing bioinformatics techniques across 33 cancer types. Our analysis encompassed XRCC1 expression levels, prognostic and diagnostic implications, epigenetic profiles, immune and molecular subtypes, Tumor Mutation Burden (TMB), Microsatellite Instability (MSI), immune checkpoints, and immune infiltration, leveraging data from TCGA, GTEx, CELL, Human Protein Atlas, Ualcan, and cBioPortal databases. Notably, XRCC1 displayed both positive and negative correlations with prognosis across different tumors. Epigenetic analysis revealed associations between XRCC1 expression and DNA methylation patterns in 10 cancer types, as well as enhanced phosphorylation. Furthermore, XRCC1 expression demonstrated associations with TMB and MSI in the majority of tumors. Interestingly, XRCC1 gene expression exhibited a negative correlation with immune cell infiltration levels, except for a positive correlation with M1 and M2 macrophages and monocytes in most cancers. Additionally, we observed significant correlations between XRCC1 and immune checkpoint gene expression levels. Lastly, our findings implicated XRCC1 in DNA replication and repair processes, shedding light on the precise mechanisms underlying its oncogenic effects. Overall, our study highlights the potential of XRCC1 as a prognostic and immunological pan-cancer biomarker, thereby offering a novel target for tumor immunotherapy.

## INTRODUCTION

Cancer represents a significant global public health challenge and is poised to surpass cardiovascular disease as the primary cause of non-natural mortality in many countries, based on prevailing year-to-year trends [1–8]. Recent reports indicate that in the year 2023, the United States is projected to witness 1,958,310 new cancer cases, with an estimated 609,820 cancer-related deaths [9]. Among the diverse spectrum of cancer types, low-grade gliomas (LGGs), classified as WHO grade II gliomas, represent a prevalent form of primary intracranial tumors, accounting for approximately 15-25% of all gliomas [10]. The annual incidence of LGGs is estimated to be around 1 case per 10,000 individuals. Notably, these tumors exhibit diffuse infiltration, gradual growth, and substantial genetic and transcriptional heterogeneity [11, 12]. LGGs are less aggressive and have a better prognosis than their high-grade counterparts, nonetheless, they remain incurable and impart significant negative impacts on patients' quality of life [13, 14]. Although conventional treatment approaches for cancer encompass surgical intervention, radiotherapy, and chemotherapy, recent advancements have brought forth the exploration of immunotherapies, targeted therapies, and epigenetic interventions [15–19]. Despite the expanding array of therapeutic modalities available to oncology patients, challenges such as tumor recurrence following surgical resection and the limited responsiveness of most patients to emerging therapies or development of treatment resistance persist, presenting significant hurdles in cancer management [20, 21]. Consequently, there is an urgent imperative to actively pursue alternative therapeutic targets and novel, highly sensitive tumor biomarkers, with the aim of unveiling innovative treatment strategies.

Sustained DNA damage in cells triggers repair pathways for cell survival, and the sort of DNA damage determines which repair pathways are activated [22]. X-ray repair cross-complementing 1 (XRCC1), a vital DNA repair scaffold, interacts closely with DNA ligase IIIα (Lig-III) to orchestrate the mending of DNA single-strand breaks triggered by ionizing radiation and alkylating agents, encompassing the base excision repair (BER) and single-strand break repair (SSBR) pathways [23, 24]. The BER pathway maintains genomic integrity and stability [25], with XRCC1 playing a critical role as a DNA repair gene by acting as a backbone protein in the initial and late steps of this pathway [26]. XRCC1 exhibits differential expression patterns across various normal human tissues, with slightly elevated levels observed in gonadal tissues and comparatively lower levels in pancreatic tissues [27]. In the context of tumorigenesis, XRCC1 manifests distinct expression profiles in different cancer tissues and serves as a valuable biological marker for tumors. Notably, nuclear expression levels of XRCC1 demonstrate associations with the prognosis of patients with biliary tract cancer and provide predictive insights into therapeutic responses following chemotherapy [28]. Intriguingly, gliomas exhibit reduced XRCC1 expression compared to normal tissues and augmented XRCC1 expression significantly impedes malignant biological behaviors [29]. Noteworthy regulatory mechanisms governing XRCC1 functionality include epigenetic modifications, gene mutations, and phosphorylation alterations. Methylation events involving XRCC1 have been linked to sperm chromatin condensation in males and sperm DNA fragmentation in patients with idiopathic oligospermia [30]. Gastric cancer tissues exhibit substantially higher levels of XRCC1 gene promoter methylation compared to adjacent normal tissues, with the hypermethylation status of this gene promoter significantly associated with protein expression loss [31]. Impairment in phosphorylation sites T358 and T367 on XRCC1 hampers its recruitment to DNA damage sites [32]. Additionally, phosphorylation of XRCC1 activates the microhomology-mediated end joining (MMEJ) pathway involved in DNA double-strand break repair, and MMEJ has been implicated in genomic rearrangements and oncogenic transformations [33].

In this study, we aimed to perform a comprehensive pan-cancer analysis of XRCC1, a pivotal gene in DNA repair, which has not been reported previously. To achieve this, we utilized multiple databases, including CCLE, CPTAC, HPA, GEO, TCGA, cBioPortal, TISIDB, TIMER, and XCELL. Our analysis aimed to examine XRCC1 expression levels across diverse cancer types and elucidate its prognostic implications. Furthermore, we explored the potential connections between XRCC1 expression and DNA methylation patterns, tumor mutational load (TMB), microsatellite instability (MSI), and immune infiltration levels in various cancer contexts. Through XRCC1 coexpression analysis and enrichment analysis, we sought to unravel the biological functions of XRCC1 in tumorigenesis. Remarkably, our findings identified XRCC1 as a novel prognostic and immunological biomarker, holding promise as a molecular target in numerous cancer types, with particular significance in LGGs. This analysis represents a rare and comprehensive approach in cancer research, harnessing genomic data from multiple cancer types to yield more comprehensive and precise outcomes.

## MATERIALS AND METHODS

### Patient data sets

To acquire the required RNAseq data, we accessed UCSC XENA (https://xenabrowser.net/datapages/) and retrieved the data in TPM (transcripts per million reads) format, which had undergone uniform processing by the Toil process [34]. For the TCGA and GTEx datasets, we performed log2 transformation on the TPM data to facilitate subsequent analysis and comparison. To conduct ROC (Receiver Operating Characteristic) analysis of XRCC1, we employed the pROC package (v.1.17.0.1) and ggplot2 package (v3.3.3) in R (v.3.6.3). Additionally, we obtained cell line gene expression matrices of tumors from the CCLE dataset (https://portals.broadinstitute.org/ccle/about). All the aforementioned analyses were implemented using the ggplot2 package (v3.3.3) in R (v4.0.3).

### Human Protein Atlas analysis

We utilized the Human Protein Atlas (https://www.proteinatlas.org/) as a valuable resource for accessing proteomic and transcriptomic data encompassing diverse human samples, including cellular, tissue, and pathological profiles. Leveraging this online database, we investigated the expression patterns of the XRCC1 gene across a range of tumor types.

### Survival analysis

To analyze RNA-seq expression data from a substantial number of tumor and normal samples, we employed the GEPIA database (http://gepia.cancer-pku.cn/) [35]. This online resource encompasses a vast collection of 9736 tumor samples and 8587 normal samples obtained from the TCGA and GTEx projects. Utilizing the “Survival Map” module within GEPIA, we accessed the OS (Overall Survival) and DFS (Disease-Free Survival) significance map data for XRCC1 across a broad spectrum of human cancers. The expression thresholds of cutoff-high (50%) and cutoff-low (50%) were utilized to stratify samples into high-expression and low-expression groups. Hypothesis testing was conducted using the log-rank test, and survival plots were obtained through the “Survival Analysis” module of GEPIA. Furthermore, we performed univariate Cox regression analysis of XRCC1 expression, specifically evaluating progression-free survival (PFS) and disease-specific survival (DSS) in tumor patients. The “forest plot” R package was employed to present P-values, hazard ratios (HR), and 95% confidence intervals (CI) for each variable of interest.

### UALCAN database analysis

The UALCAN database (http://ualcan.path.uab.edu/analysis-prot.html) [36] offers an interactive platform for conducting comprehensive analyses of TCGA gene expression data. Within this database, we utilized the CPTAC dataset [37] to examine protein expression and phosphoprotein levels. Furthermore, we employed the UALCAN database's coverage of the TCGA dataset to investigate the methylation levels of XRCC1 DNA across various cancer types. Statistical analysis was conducted using Student's t-test to assess the significance of differences, with a p-value threshold of less than 0.05 considered statistically significant.

### Pan-cancer analysis of genetic alterations of XRCC1

The cBio Cancer Genomics Portal (cBioPortal) (http://cbioportal.org) [38] is a valuable resource for exploring, visualizing, and analyzing multidimensional cancer genomics data. In our study, we leveraged the cBioPortal database to investigate the mutation frequency, mutation types, copy number alterations (CNAs), and mutation sites of XRCC1 across all TCGA tumors. Furthermore, we examined the potential relationship between genetic alterations in XRCC1 and prognosis in patients with different cancer types. Additionally, TMB and MSI scores were obtained from the TCGA dataset, and we performed correlation analysis, employing Spearman's rank correlation coefficient, to assess the association between XRCC1 expression and TMB as well as MSI.

### TISIDB database analysis

The TISIDB database (http://cis.hku.hk/TISIDB/index.php) [39] serves as a comprehensive web portal focusing on tumor and immune system interactions, encompassing diverse data types. In our investigation, we utilized the TISIDB database to explore the correlations between XRCC1 expression and immune or molecular subtypes in various cancer types. Statistical significance was determined with a p-value threshold of less than 0.05, indicating meaningful differences in the observed associations.

### Correlation analysis of XRCC1 expression with immune infiltrating cells and their marker genes

We obtained data for 33 cancers and normal tissues from the Genomic Data Commons (GDC) [40] data portal within the TCGA database. To ensure an accurate assessment of immune correlations, we utilized the immunedeconv R package, which integrates six advanced algorithms: TIMER, XCELL, CIBERSORT, EPIC, MCPCOUNTER, and QUANTISEQ. The generated heat map depicts different types of cancer on the horizontal axis, various immune scores on the vertical axis, and correlation coefficients represented by distinct colors. Additionally, we explored the relationship between XRCC1 expression in immune cells and multiple markers using the TIMER database (http://cistrome.org/TIMER/) [41]. The x-axis displays XRCC1 expression levels, while the y-axis represents other relevant genes. Statistical significance is denoted by asterisks: * indicates p < 0.05, ** indicates p < 0.01, and *** indicates p < 0.001.

### Correlation analysis of XRCC1 expression with immune checkpoint

To delve deeper into the analysis of the expression of 47 common immune checkpoint genes in relation to XRCC1, we employed the limma package [42]. Specifically, we extracted these immune checkpoint genes and calculated their correlation with XRCC1 expression individually. For a correlation to be considered significant, we set the thresholds at p < 0.05 and R > 0.20, indicating a statistically significant positive correlation between XRCC1 and the immune checkpoint genes.

### Gene enrichment analysis and PPI network construction

We explored the differential expression genes (DEGs) between different XRCC1 expression groups (low expression group: 0–50%; high expression group: 50–100%) in LGG using the DESeq2 package. The volcano map was drawn by the ggplot2 package with the threshold values of |log2 fold-change (FC)|>1.0 and adjusted P-value<0.05. Then, we performed GO term enrichment, KEGG pathway enrichment and Gene enrichment analysis (GSEA) of DEGs using the ggplot2 package for visualization and the clusterProfiler package [43] for statistical analysis. The Tool for Interactive Gene Search (STRING) (https://string-db.org/cgi/input.pl) [44] is a public database providing protein interaction information. We used the STRING online database to construct PPI networks for 134 genes obtained at threshold |log2 fold-change (FC)|>2.0 and adjusted p-value<0.05 and screened hub gene by Cytoscape [45].

### Analysis of mutations, methylation, and pathway activity of XRCC1 and its hub gene

To further expand our analysis, we utilized GSCALite (http://bioinfo.life.hust.edu.cn/web/GSCALite/) [46], which offers various analysis modules to examine multi-omics data. The database incorporates data from multiple sources, including 11,160 samples from TCGA covering 33 cancer types (TCGA Cancer), information on Cancer Drug Sensitivity Genomics with 746 drug data from the Cancer Therapeutic Response Portal, as well as 11,688 normal tissue expression data from the Genotype-Tissue Expression (GTEx) project. With the help of GSCALite, we investigated several aspects, including copy number variation (CNV), methylation patterns, pathway activity, and drug sensitivity. These analyses allow us to gain insights into the genetic alterations, epigenetic modifications, cellular pathway dynamics, and potential therapeutic responses associated with XRCC1 and its involvement in different cancers.

### Statistical analysis

In our study, R (version 3.6.3) was employed for conducting statistical analyses. The Wilcoxon signed rank sum test was utilized to investigate the expression of XRCC1 in tumor tissues and their corresponding neighboring tissues. Cox regression analysis and the Kaplan-Meier method were applied to evaluate prognostic factors. Student's test was used to compare protein expression, phosphoprotein levels, and DNA methylation levels of XRCC1 between normal and tumor groups. The correlation between XRCC1 expression and tumor mutational burden (TMB) as well as microsatellite instability (MSI) was examined using Spearman's rank correlation analysis. Furthermore, we assessed the association between XRCC1 expression and clinical-pathological characteristics of LGG using the Kruskal-Wallis and Wilcoxon rank sum tests. A p-value below 0.05 was considered statistically significant in all statistical tests performed.

### Data availability statement

The datasets presented in this study can be found in online repositories. The names of the repository/repositories and accession number(s) can be found in the article Supplementary Material.

## RESULTS

### Pan-cancer analysis of XRCC1 differential expression and clinicopathological correlation

**Figure 1:**
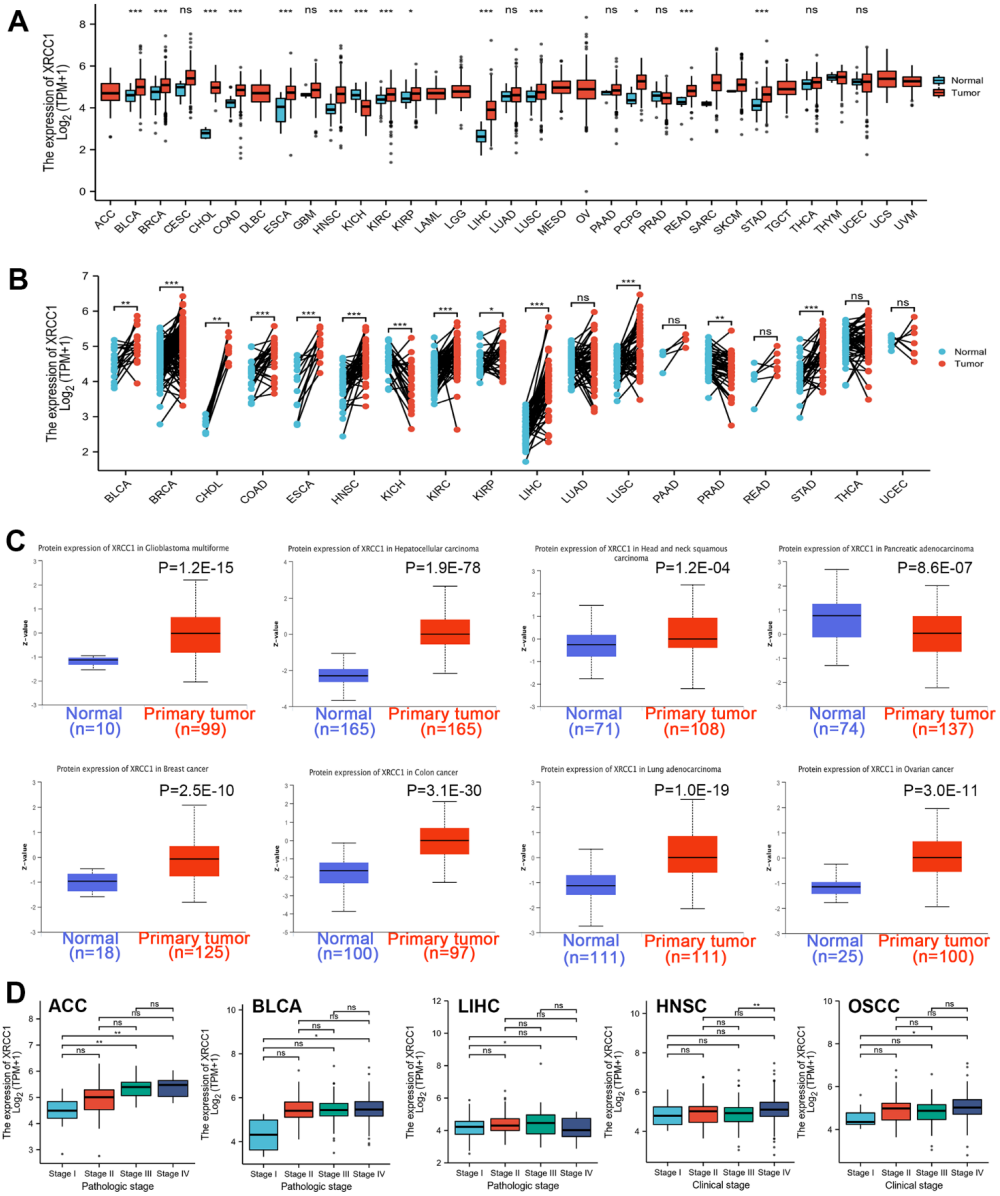
(**A**) Human XRCC1 gene expression status in various cancers from TCGA database. (**B**) Wilcoxon signed rank sum test was used to detect the differential expression of XRCC1 in tumor tissues and adjacent paracancerous tissues. (**C**) The protein expression level of the XRCC1 gene in glioblastoma multiforme, hepatocellular carcinoma, head and neck squamous carcinoma, pancreatic adenocarcinoma, breast cancer, ovarian cancer, colon cancer, lung adenocarcinoma, clear cell RCC. (**D**) The box plot of tumor pathological stages (stage I, stage II, stage III, stage IV) following ACC, BLCA, LIHC, and clinical stages (stage I, stage II, stage III, stage IV) of HNSC, OSCC. ns P≥0.05, *P<0.05, **P<0.01; ***P<0.001.

In this study, we aimed to investigate the role of XRCC1 in carcinogenesis. Firstly, we analyzed the expression status of XRCC1 in various tumor tissues and paired normal tissues (Figure 1A). The results revealed significantly higher expression levels of XRCC1 in tumor tissues compared to normal tissues in multiple cancer types, including bladder urothelial carcinoma (BLCA), breast invasive carcinoma (BRCA), cholangiocarcinoma (CHOL), colon adenocarcinoma (COAD), esophageal carcinoma (ESCA), head and neck adenocarcinoma (HNSC), kidney renal clear cell carcinoma (KIRC), kidney renal papillary cell carcinoma (KIRP), liver hepatocellular carcinoma (LIHC), lung squamous cell carcinoma (LUSC), pheochromocytoma and paraganglioma (PCPG), rectum adenocarcinoma (READ), and stomach adenocarcinoma (STAD). Conversely, the expression level of XRCC1 was low in the kidney chromophobe (KICH) (Figure 1A). We further validated our findings by analyzing the expression of XRCC1 in normal samples from the Genotype-Tissue Expression (GTEx) database and matched adjacent tumor tissues (Supplementary Figure 1A). To assess the expression of XRCC1 in tumor tissues compared to their matched adjacent tissues, we performed the Wilcoxon signed rank sum test (Figure 1B). Moreover, we explored the expression of XRCC1 in a broader range of cancer tissues using the Cancer Cell Line Encyclopedia (CCLE) database, which provided cell line expression matrices for various tumor types (Supplementary Figure 1B). To examine the protein expression of XRCC1, we utilized the Clinical Proteomic Tumor Analysis Consortium (CPTAC) database. Our analysis demonstrated significantly higher protein expression levels of XRCC1 in primary tissues of GBM, hepatocellular carcinoma, head and neck squamous carcinoma, breast cancer, colon cancer, lung adenocarcinoma (LUAD), and ovarian cancer compared to normal tissues (Figure 1C). Additionally, we provided supplemental protein expression data for XRCC1 genes that did not show significant differences (Supplementary Figure 1C). These findings were further confirmed using the Human Protein Atlas (HPA) database (Supplementary Figure 2). Finally, we investigated the association between XRCC1 gene expression and clinical characteristics. We assessed XRCC1 expression levels in different tumor stages of Adrenocortical carcinoma (ACC), BLCA, and LIHC. Furthermore, we examined the correlation between XRCC1 expression and clinical stages in head and neck squamous cell carcinoma (HNSC) and oral squamous cell carcinoma (OSCC) (Figure 1D). Additionally, we analyzed the differential expression of XRCC1 based on patient age, gender, T-stage, and histological grade (Supplementary Figure 3).

### Pan-cancer analysis of the multifaceted prognostic value of XRCC1

**Figure 2:**
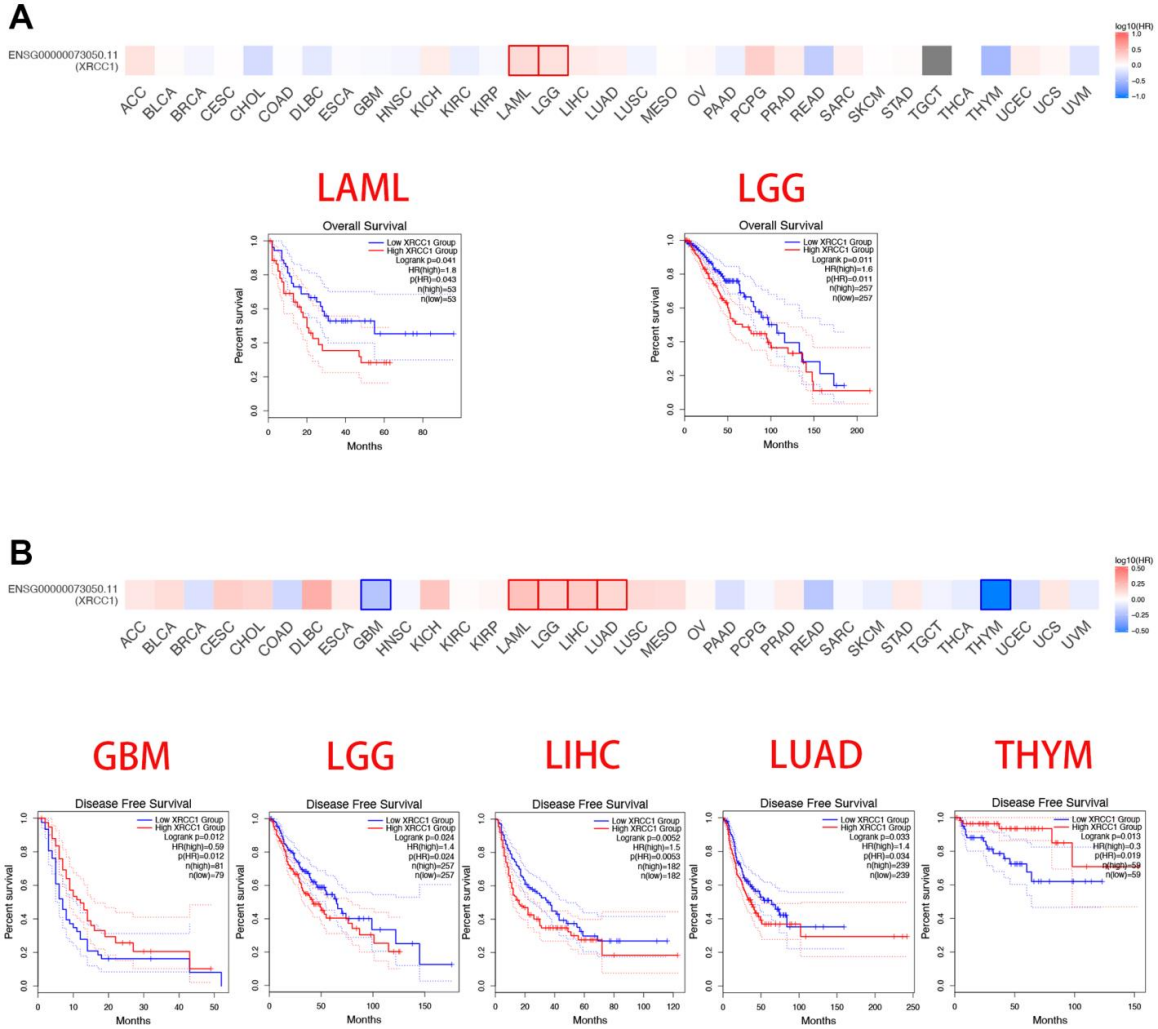
**Summary of relativity between survival prognosis across cancer types in TCGA dataset and XRCC1 gene expression levels.** The GEPIA database was utilized to plot overall survival (**A**) and disease-free survival (**B**) conditions across cancer types in TCGA dataset by XRCC1 gene expression. We observed that high XRCC1 gene expression was related to worse OS and DFS in almost all cancer types, except DFS in UCEC cohorts(n=160) and THYM cohorts(n=118). Only p-values < 0.05 were displayed.

**Figure 3:**
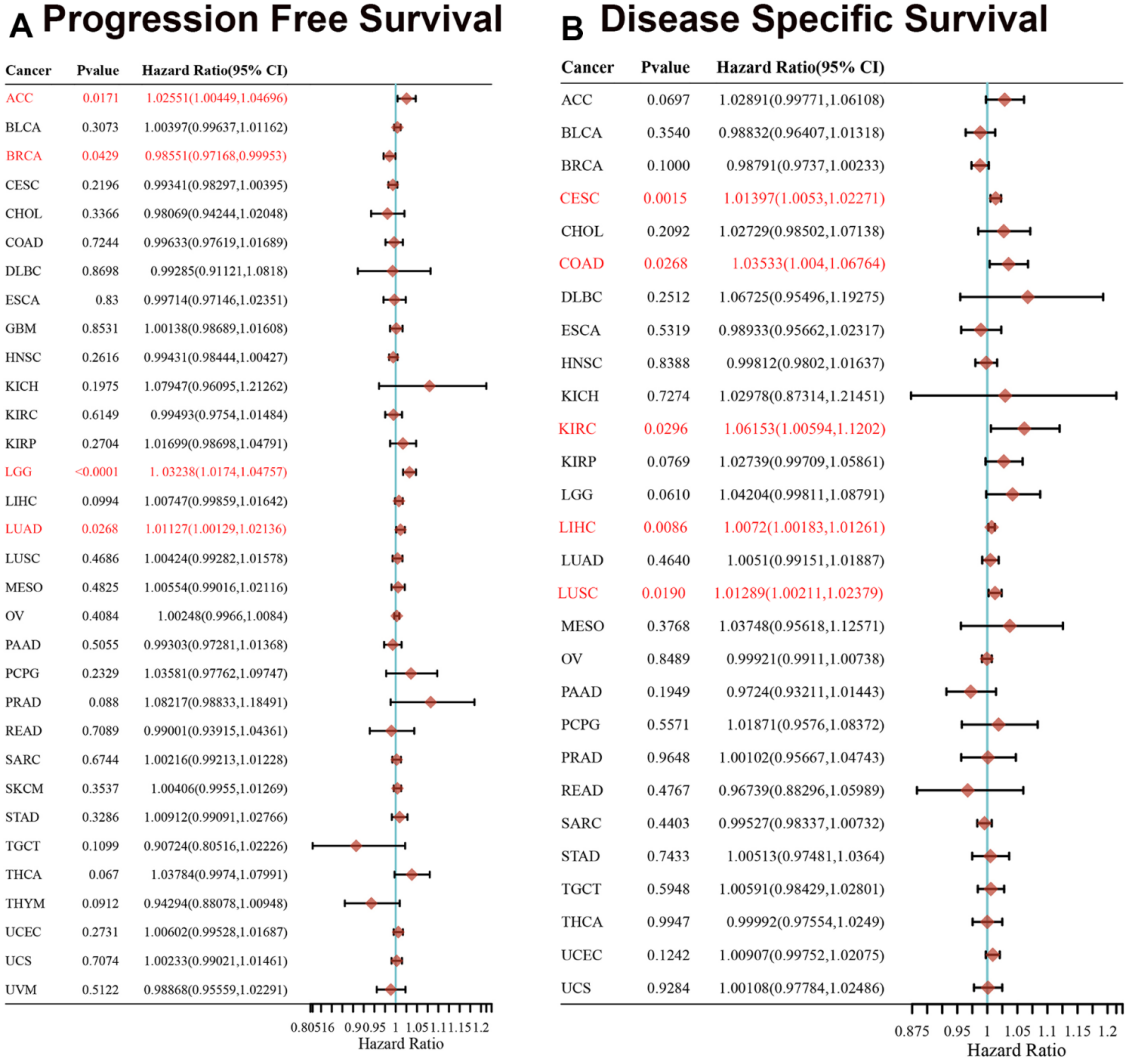
**Association between XRCC1 expression levels and cancer patients' PFS and DSS prognosis, using univariate survival analysis in various cancers.** (**A**) Relevance of XRCC1 gene expression levels to PFS prognosis across cancer types in TCGA dataset. (**B**) Relevance of XRCC1 gene expression to DSS prognosis across cancer types in TCGA dataset. Red font indicated P-value < 0.05.

We separated the cancer cases into low-expression and high-expression groups based on the gene expression levels of XRCC1. We explored the relativity between XRCC1 gene expression levels and the survival prognosis across cancer types with the assistance of GEO and TCGA datasets. According to Figure 2A, high XRCC1 gene expression was related to poor overall survival (OS) in cancers of acute myeloid leukemia (LAML) (n=106, P=0.041) and LGG (n=514, P=0.011) in the TCGA dataset. Disease-free survival (DFS) relevant statistics in Figure 2B revealed a linkage between poor prognosis and high XRCC1 gene expression levels for the cancer cases from the TCGA dataset of LGG (n=521, P=0.024), LIHC (n=364, P=0.0053) and LUAD (n=478, P=0.034). Meanwhile, high expression of the XRCC1 gene was linked to better DFS in GBM (n=160, P=0.012) and thymoma (THYM) (n=118, P=0.019). In summary, we found that the gene expression level of XRCC1 was mainly relevant to the poor survival prognosis of patients with various cancer types. Furthermore, we conducted a survival relevance analysis across multiple cancer types, including progression-free survival (PFS) and disease-specific survival (DSS), to learn the relevance of XRCC1 gene expression levels to the survival prognosis of cancer patients. The forest plot of PFS analysis indicated that higher XRCC1 gene expression levels were relevant to worse PFS in ACC (P=0.0171), LGG (P <0.0001) and LUAD (P= 0.0268), whereas better PFS in BRCA(P=0.0429) (Figure 3A). Furthermore, analysis of DSS prognosis (Figure 3B) showed the relevance of higher XRCC1 gene expression levels to worse DSS in patients with cervical squamous cell carcinoma and endocervical adenocarcinoma (CESC) (P=0.0015), COAD (P=0.0268), KIRC (P=0.0296), LIHC (P=0.0086) and LUSC (P=0.0190). The data from other cancer types are all statistically insignificant. In summary, our findings indicate that XRCC1 gene expression levels are predominantly associated with poor survival prognosis in various cancer types. Furthermore, the analysis of PFS and DSS across multiple cancer types further supports the relevance of XRCC1 gene expression levels to the survival prognosis of cancer patients.

### Diagnostic value of XRCC1 in pan-cancer

**Figure 4:**
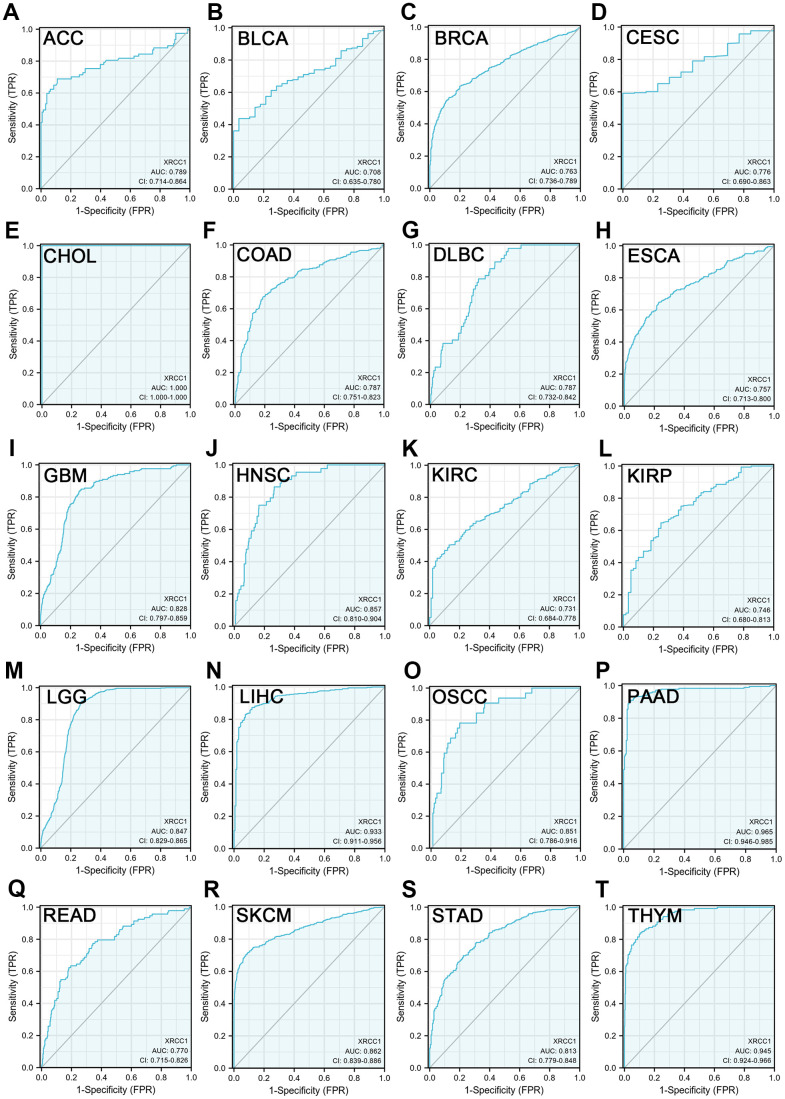
**XRCC1 expression levels could distinguish between cancerous and normal tissues in pan-cancer.** (**A**) ACC; (**B**) BLCA; (**C**) BRCA; (**D**) CESC; (**E**) CHOL; (**F**) COAD; (**G**) DLBC; (**H**) ESCA; (**I**) GBM; (**J**) HNSC; (**K**) KIRC; (**L**) KIRP; (**M**) LGG; (**N**) LIHC; (**O**) OSCC; (**P**) PAAD; (**Q**) READ; (**R**) SKCM; (**S**) STAD; (**T**) THYM. X-axis reveals the false positive rate, while Y-axis indicates the true positive rate.

In our analysis, we utilized receiver operating characteristic (ROC) curves to assess the predictive ability of XRCC1 expression levels in distinguishing between cancer and normal tissue across multiple cancer types. The results demonstrated that XRCC1 exhibited a considerable accuracy (AUC>0.7) in predicting 20 cancer types, including ACC (AUC=0.789), BLCA (AUC=0.708), BRCA (AUC=0.763), CESC (AUC=0.776), CHOL (AUC=1.000), COAD (AUC=0.787), DLBC (AUC=0.787), ESCA (AUC=0.757), GBM (AUC=0.828), HNSC (AUC=0.857), KIRC (AUC=0.731), KIRP (AUC=0.746), LGG (AUC=0.847), LIHC (AUC=0.933), OSCC (AUC=0.851), PAAD (AUC=0.965), READ (AUC=0.770), SKCM (AUC=0.862), STAD (AUC=0.813), and THYM (AUC=0.945) (Figure 4). Notably, XRCC1 exhibited high accuracy (AUC>0.9) in predicting CHOL, LIHC, PAAD, and THYM. These findings suggest that XRCC1 has the potential to serve as an excellent biomarker for cancer diagnosis. Supplementary Figure 4 provides the ROC curves for XRCC1 in other cancer types for reference.

### Pan-cancer analysis of the phosphorylation of XRCC1

**Figure 5:**
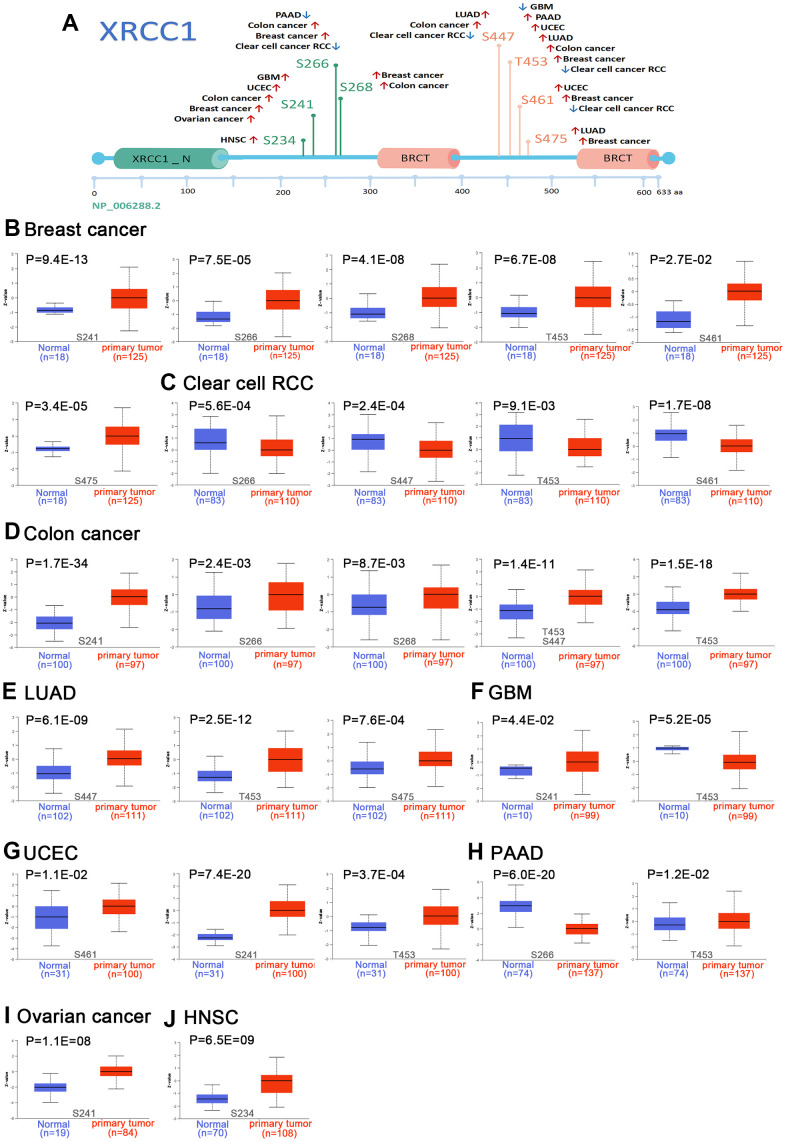
**We utilized the UALCAN tool to compare the expression status of XRCC1 phosphoprotein (NP_006288.2, S234, S241, S266, S268, S447, S461, S475, and T453 sites) between primary tissue of specific tumors and normal tissue from CPTAC database.** The schematic diagram of XRCC1 protein (**A**) demonstrated the phosphoprotein sites with positive results. We also enriched the box plots with different tumors containing breast cancer (**B**), clear cell RCC (**C**), COAD (**D**), LUAD (**E**), GBM (**F**), UCEC (**G**), PAAD (**H**), ovarian cancer (**I**), HNSC (**J**).

In our analysis, we investigated the differences in XRCC1 protein expression and phosphorylation between primary tumor tissues and normal tissues. Utilizing the CTPAC database, we focused on nine types of tumors, namely breast cancer, ovarian cancer, COAD, RCC, UCEC, LUAD, GBM, PAAD, and HNSC. Initially, we examined the XRCC1 protein phosphorylation sites and identified notable differences (Figure 5A). Specifically, the phosphorylation level at the T453 locus within the XRCC1 protein was significantly elevated in breast cancer, colon cancer, LUAD, UCEC, and pancreatic adenocarcinoma compared to normal tissues (all P < 0.05) (Figure 5B, 5D, 5E, 5G, 5H). Furthermore, we observed an increased phosphorylation level at the S241 locus within XRCC1 protein in breast cancer (P = 9.4E-13), colon cancer (P = 1.7E-34), GBM (P = 4.4E-02), UCEC (P = 7.4E-20), and ovarian cancer (P = 1.1E-08) (Figure 5B, 5D, 5F, 5G, 5I). These findings indicate the potential significance of phosphorylation events in tumorigenesis. Further molecular assays are warranted to gain a deeper understanding of the role of phosphorylation at these specific loci in various types of cancer development.

### Pan-cancer analysis of the methylation level and genetic alteration of XRCC1

**Figure 6:**
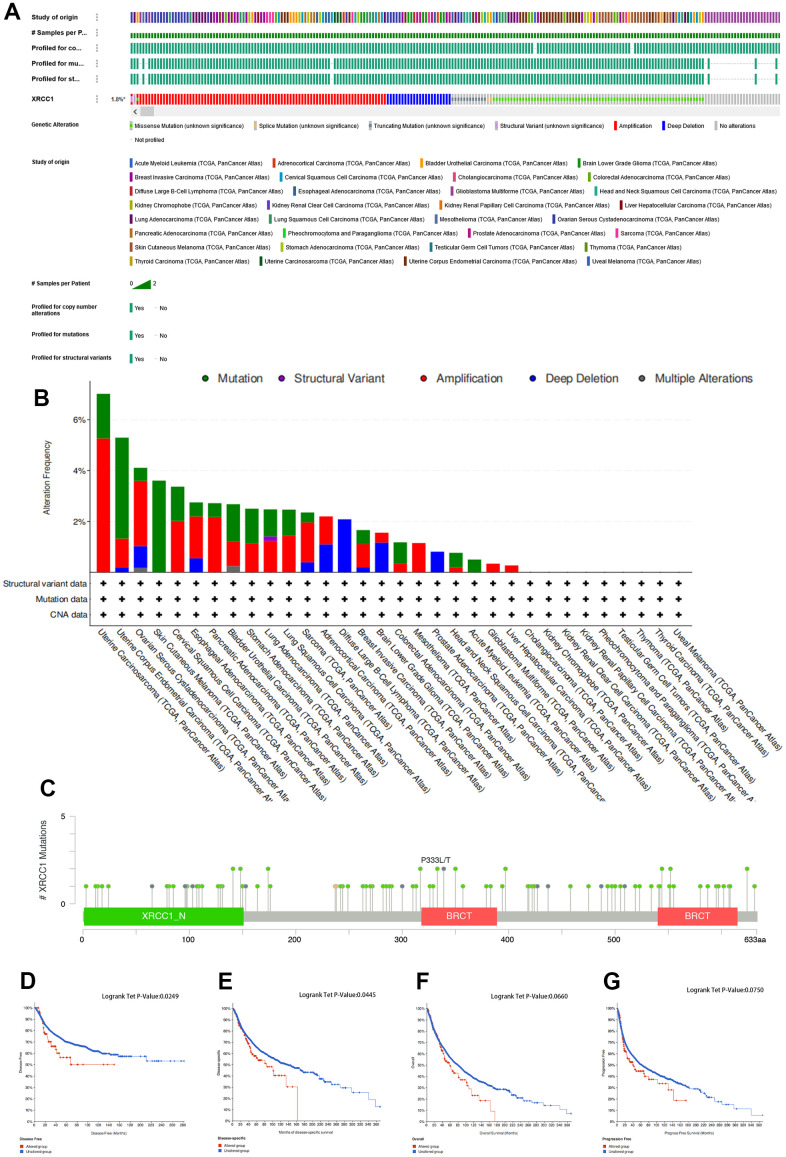
**Mutation feature of XRCC1 in pan-cancer of TCGA.** (**A**) The cBioPortal database was used to analyze the proportion of patients with XRCC1 genomic alterations in pan-cancer. The frequency of mutation type (**B**) and mutation site (**C**) of XRCC1 in TCGA tumors was analyzed using the cBioPortal tool. The cBioPortal database was used to explore the impact of XRCC1 mutation status on OS (**D**), DFS (**E**), PFS (**F**), and DSS (**G**) of cancer patients.

**Figure 7:**
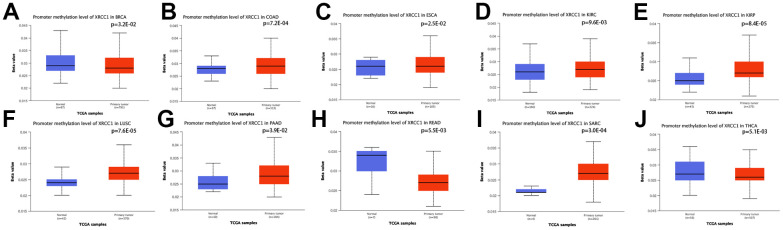
**Promoter methylation level of XRCC1 in pan-cancer.** (**A**) in BRCA, (**B**) in COAD, (**C**) in ESCA, (**D**) in KIRC, (**E**) in KIRP, (**F**) in LUSC, (**G**) in PAAD, (**H**) in READ, (**I**) in SARC, (**J**) in THCA.

We conducted an analysis of XRCC1 genomic alterations in pan-cancer using the cBioPortal (TCGA, The Pan-Cancer Atlas) database. Our findings revealed that genomic alterations in XRCC1 were detected in 1.8% of patients (Figure 6A). Further exploration of the genetic alteration status of XRCC1 in different tumor samples within the TCGA cohort showed distinct patterns. Among these, the highest frequency of alterations in XRCC1 (>6%) was observed in patients with uterine carcinosarcoma (UCS), where “Amplification” was the predominant type of alteration (Figure 6B). In cases of DLBC with genetic alterations, copy number deletion of XRCC1 was the prevalent type of alteration. Notably, the “Structural Variant” type of XRCC1 alteration was exclusively found in cases of LUSC. Furthermore, we analyzed the types, sites, and cases of XRCC1 genetic alterations. Missense mutations were the most common type of alteration, and specific alterations such as P333L/T in the BRCT structural domain were identified in OV and ESCA cases (Figure 6C). Additionally, we investigated the potential association between XRCC1 genetic alterations and patient prognosis across different cancer types. The results demonstrated that patients with tumors harboring XRCC1 gene alterations exhibited poorer disease-free survival (DFS) and disease-specific survival (DSS) compared to those without alterations. Although overall survival (OS) and progression-free survival (PFS) did not reach statistical significance in the analysis between the two groups, a similar trend was observed (Figure 6D). Furthermore, we utilized the UALCAN database to analyze XRCC1 DNA methylation levels. The analysis revealed significant reductions in XRCC1 DNA methylation levels in COAD, ESCA, KIRC, KIRP, LUSC, PAAD, and SARC tissues compared to normal tissues. Conversely, elevated levels of XRCC1 DNA methylation were observed in BRCA and READ (Figure 7A). However, no significant differences in XRCC1 DNA methylation levels were observed in other tumor tissues compared to their matched normal tissues (Supplementary Figure 5).

### Pan-cancer analysis of TMB and MSI of XRCC1

**Figure 8:**
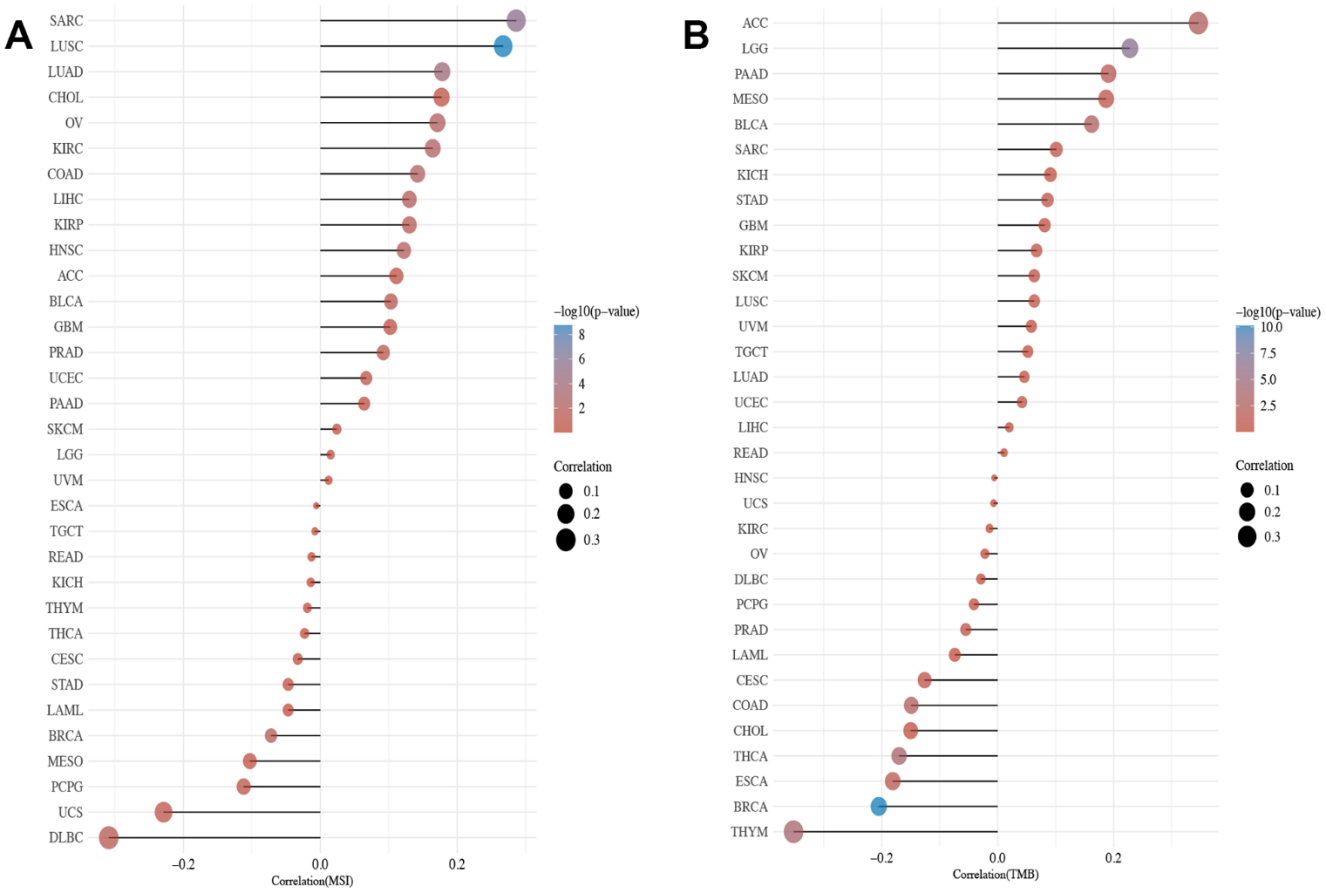
**The relevance of XRCC1 gene expression levels to TMB and MSI across cancer types.** (**A**) A bar chart reveals the relevance of XRCC1 gene expression levels to TMB in pan-cancer. (**B**) A bar chart shows the relationship between XRCC1 gene expression levels and MSI in multiple cancer types. The horizontal axis in the figure represents the correlation coefficient between genes and TMB/MSI, the ordinate is different tumors, the size of the dots in the figure represents the value of the correlation coefficient, and the different colours represent the degree of statistical significance. The bluer the colour, the smaller the p-value.

The study also investigated the association between XRCC1 gene expression levels and two significant biomarkers related to immunotherapy response: tumor mutational burden (TMB) and microsatellite instability (MSI). The results indicated that XRCC1 gene expression levels were significantly correlated with TMB status in several cancer types, including SARC, LUSC, UCS, and DLBC. Among these, LUSC showed particularly strong statistical significance (Figure 8A), suggesting a potential role of XRCC1 in modulating TMB in this cancer type. Furthermore, the relationship between XRCC1 gene expression levels and MSI status was explored across multiple human cancers. The analysis revealed positive correlations between XRCC1 expression and MSI status in ACC, LGG, PAAD, MESO, and BLCA. On the other hand, BRCA and THYM showed negative correlations between XRCC1 expression and MSI status. It is worth noting that the p-value for BRCA was smaller than that of other cancers, indicating a potentially significant association (Figure 8B). These findings suggest that XRCC1 gene expression levels may be linked to the underlying genomic instability and DNA repair processes associated with TMB and MSI in various cancer types.

### Pan-cancer analysis of immune and molecular subtypes of XRCC1

**Figure 9:**
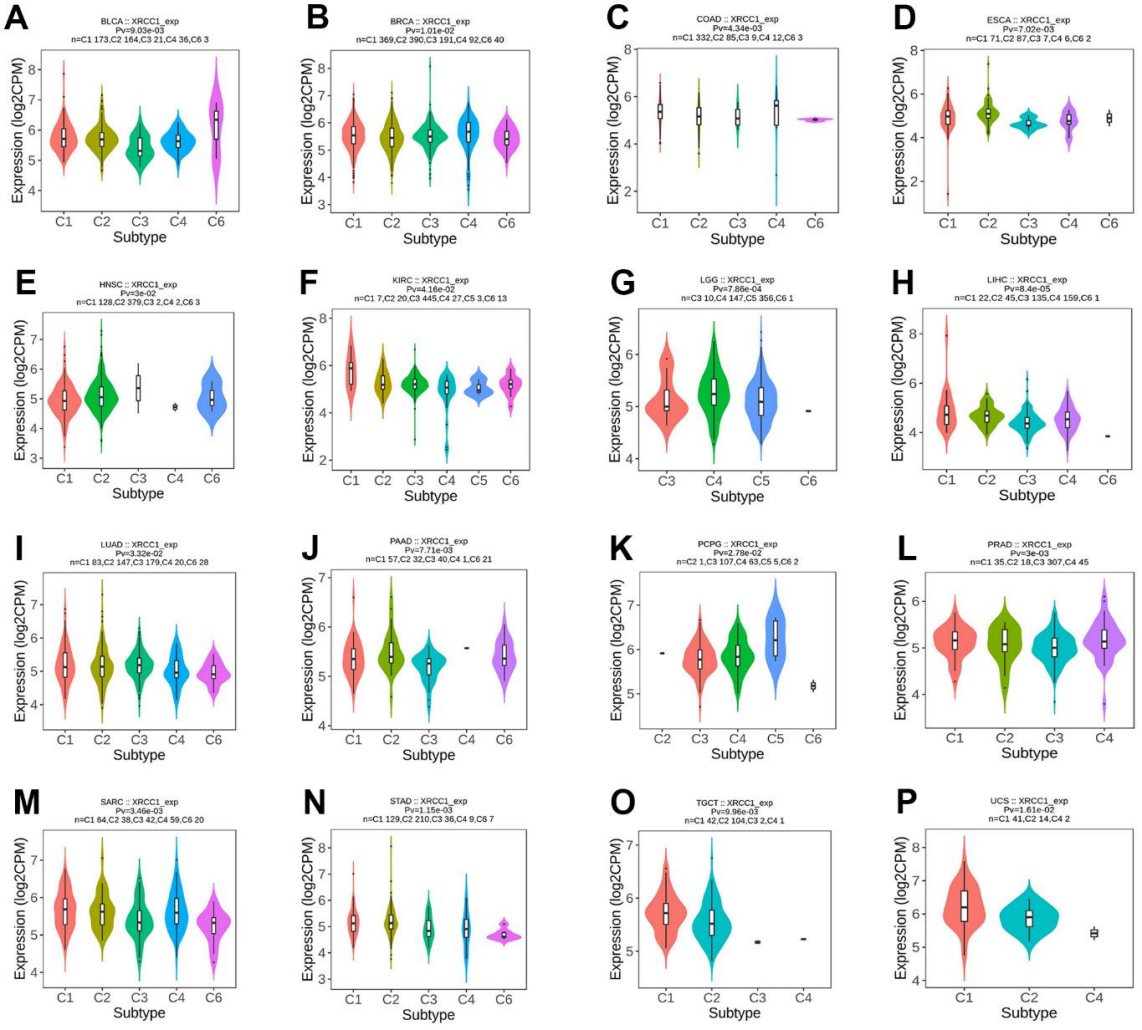
**The relationship between XRCC1 expression and pan-cancer immune subtypes.** (**A**) in BLCA, (**B**) in BRCA, (**C**) in COAD, (**D**) in ESCA, (**E**) in HNSC, (**F**) in KIRC, (**G**) in LGG, (**H**) in LIHC, (**I**) in LUAD, (**J**) in PAAD, (**K**) in PCPG, (**L**) in PRAD, (**M**) in SARC, (**N**) in STAD, (**O**) in TGCT, (**P**) in UCS.

**Figure 10:**
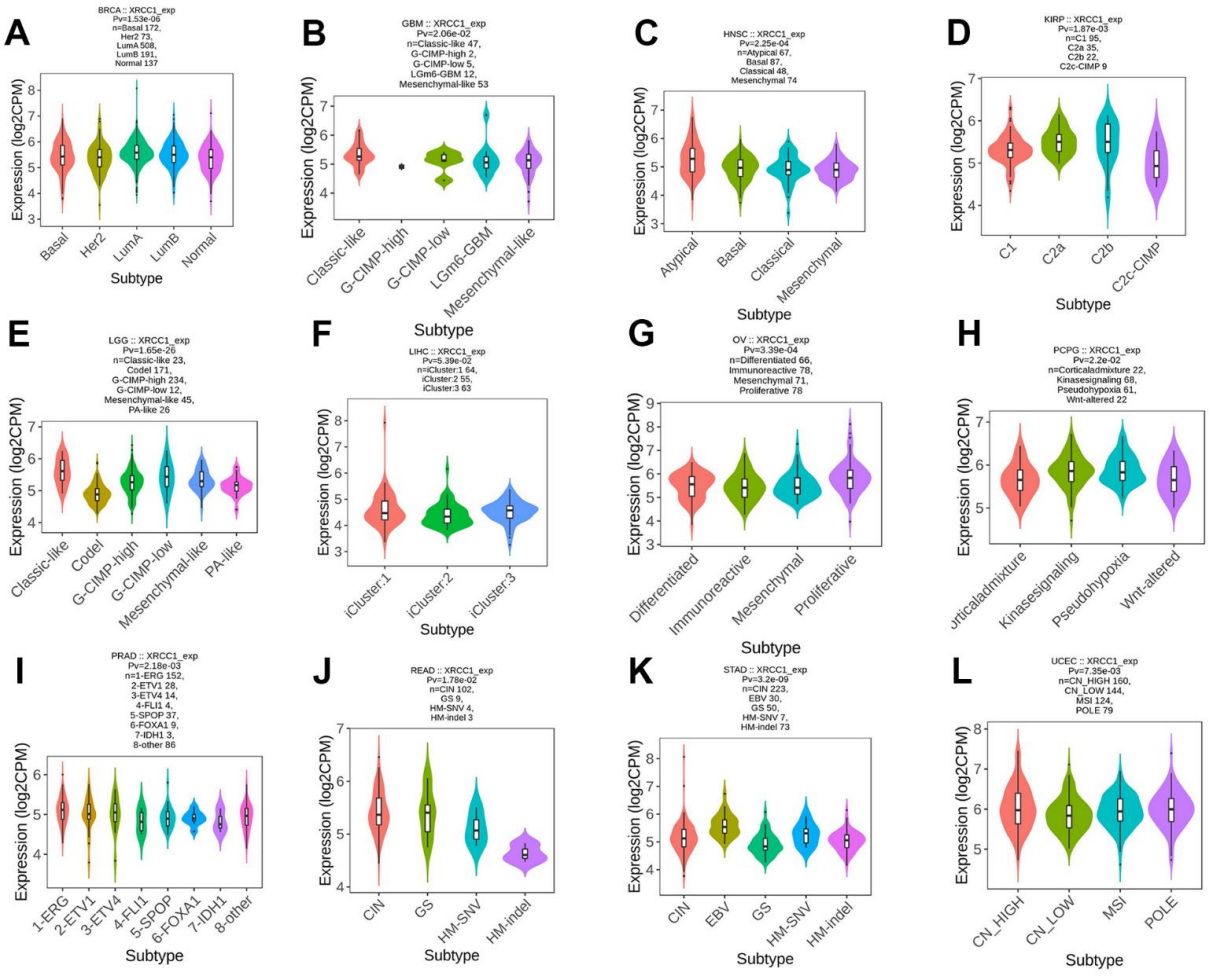
**The relationship between XRCC1 expression and pan-cancer molecular subtypes.** (**A**) in BRCA, (**B**) in GBM, (**C**) in HNSC, (**D**) in KIRP, (**E**) in LGG, (**F**) in LIHC, (**G**) in OV, (**H**) in PCPG, (**I**) in PRAD, (**J**) in READ, (**K**) in STAD, (**L**) in UCEC.

The study conducted an analysis of XRCC1 expression in relation to immune subtypes and molecular subtypes of human cancer using the subtype module of the TISIDB database. Immune subtypes were classified into six types: C1 (wound ranging), C2 (IFN-gamma dominant), C3 (inflammatory), C4 (lymphocyte depleted), C5 (immunologically quiet), and C6 (TGF-B dominant). The analysis revealed significant differences in XRCC1 expression among different immune subtypes in multiple cancer types, including BLCA, BRCA, COAD, ESCA, HNSC, KIRC, LGG, LIHC, LUAD, PAAD, PCPG, PRAD, SARC, STAD, TGCT, and UCS (Figure 9). Additionally, XRCC1 expression displayed significant differences among different molecular subtypes in several cancer types, including BRCA, GBM, HNSC, KIRP, LGG, LIHC, OV, PCPG, READ, STAD, and UCEC (Figure 10). XRCC1 expression in different immune and molecular subtypes of other cancers is shown in Supplementary Figure 6. These findings indicate that XRCC1 expression varies significantly across different immune and molecular subtypes in various human cancer types, highlighting the potential involvement of XRCC1 in distinct tumor microenvironments and molecular pathways.

### Pan-cancer analysis of the XRCC1 expression and immune cell infiltration

**Figure 11:**
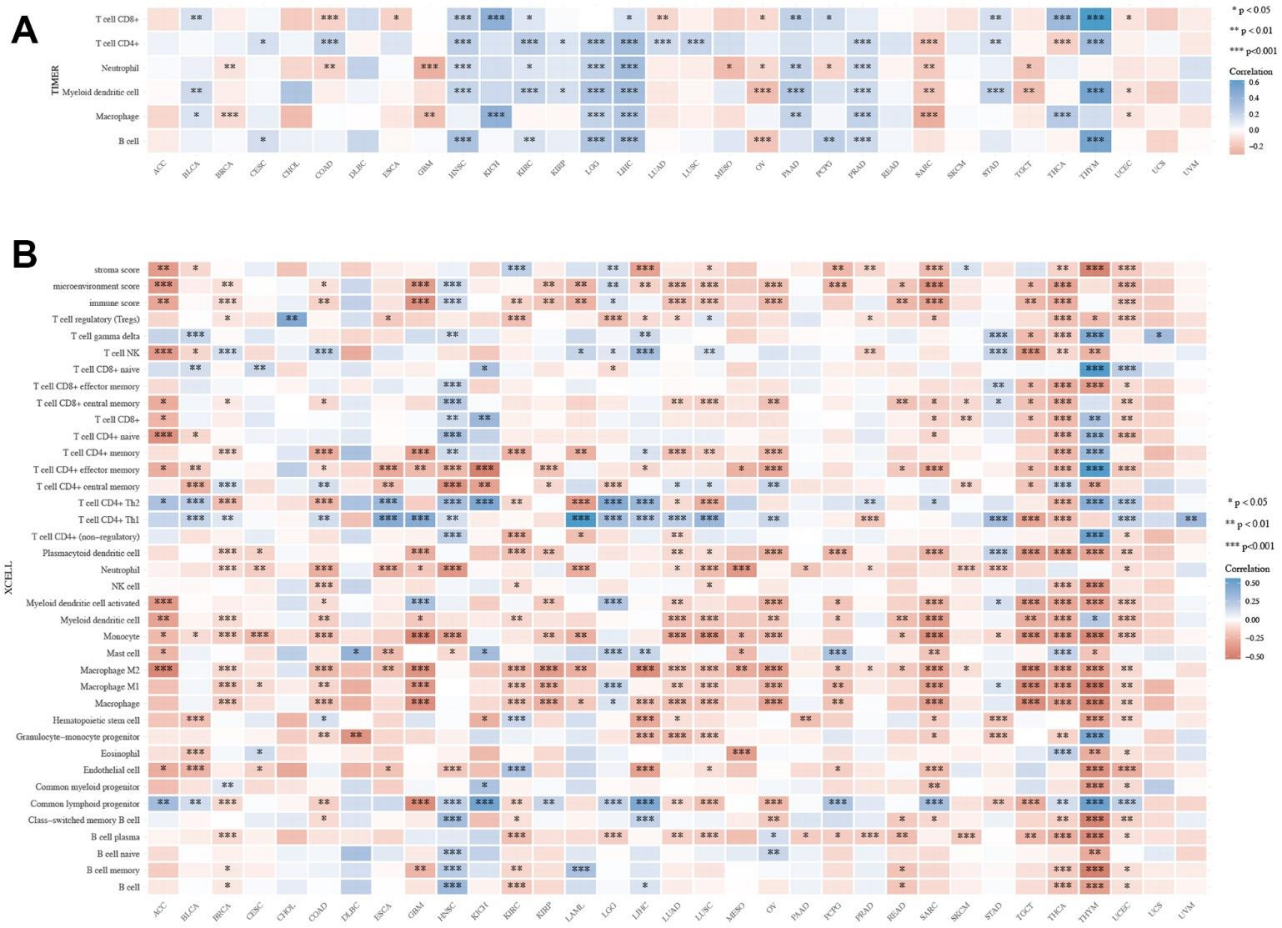
**The XRCC1 expression correlated with immune infiltration.** (**A**) The expression of XRCC1 was strongly associated with the infiltration levels of various immune cells in the TIMER dataset. (**B**) Based on the XCELL database, we explored the significant correlation between the expression of XRCC1 and the infiltration levels of various immune cells. (*p < 0.05, **p < 0.01, and ***p < 0.001).

The study utilized the TIMER and XCELL datasets to investigate the correlation between XRCC1 expression and immune infiltration in multiple tumor tissues. The results demonstrated significant correlations between XRCC1 expression and the abundance of infiltrating immune cells in various cancer types. Specifically, XRCC1 expression showed significant correlations with T cell CD8+ infiltration in 15 types of cancer, T cell CD4+ infiltration in 14 types of cancer, neutrophil infiltration in 14 types of cancer, myeloid dendritic cell infiltration in 11 types of cancer, and B cell infiltration in 9 types of cancer. The significance of these correlations was assessed using the Wilcox test (Figure 11A). Furthermore, the study employed the XCELL algorithm to explore the relationship between XRCC1 expression and the immune infiltration levels of different immune cell subtypes. The analysis revealed a significant negative correlation between XRCC1 expression and immune infiltration in LUAD, LUSC, SARC, TGCT, THCA, and UCEC. Additionally, positive associations were observed between XRCC1 expression and common lymphoid progenitor infiltration, as well as T cell CD4+ Th1 and Th2 cell infiltration, across various cancer types (Figure 11B). Similar results were obtained using other algorithms such as CIBERSORT, EPIC, MCPCOUNTER, and QUANTISEQ (Supplementary Figure 7).

Subsequently, we investigated the correlation between XRCC1 expression status and immune cell markers in LGG through the TIMER database, involving B cell, CD8+ T cells, M1/M2 macrophages, tumor-associated macrophages (TAM), neutrophils, Natural killer cell, dendritic cells. Meanwhile, a series of functional T cells, like Th1, Th2, Th9, Th17, Th22, Tfh, exhausted T cells, Treg cells were also be examined. The outcomes suggested that XRCC1 expression was obviously associated with the infiltration levels of immune cell markers containing B cell, Tfh, Th2, Th9, Th22, exhausted T cell, macrophage, TAM, monocyte, neutrophil and natural killer cell (Table 1).

**Table 1:** Correlation analysis between XRCC1 and markers of immune cells in TIMER.

**Cell type**	**Gene marker**	**None**			**Purity**	
		**Cor**	**P**		**Cor**	**P**
B cell	CD19	0.151	***		0.156	***
	CD20(KRT20)	0.007	0.88		0.016	0.72
	CD38	0.117	**		0.101	*
CD8+ T cell	CD8A	-0.051	0.25		-0.07	0.13
	CD8B	-0.101	*		-0.106	*
Tfh	BCL6	0.175	***		0.179	***
	ICOS	0.07	0.11		0.06	0.19
	CXCR5	0.131	**		0.153	***
Th1	T-bet (TBX21)	0.108	*		0.112	*
	STAT4	-0.241	***		-0.245	***
	IL12RB2	-0.079	0.07		-0.093	*
	WSX1(IL27RA)	-0.023	0.60		-0.018	0.69
	STAT1	0.154	***		0.141	**
	IFN-γ (IFNG)	0.051	0.24		0.064	0.16
	TNF-α(TNF)	0.081	0.07		0.067	0.14
Th2	GATA3	0.179	***		0.181	***
	CCR3	0.099	*		0.098	*
	STAT6	0.082	0.06		0.081	0.08
	STAT5A	0.312	***		0.333	***
Th9	TGFBR2	0.136	**		0.141	**
	IRF4	0.055	0.21		0.089	0.05
	PU.1(SPI1)	0.292	***		0.302	***
Th17	STAT3	0.275	***		0.268	***
	IL-21R	-0.123	**		-0.131	**
	IL-23R	0.004	0.94		-0.017	0.72
	IL-17A	-0.01	0.82		0.005	0.91
Th22	CCR10	0.122	**		0.139	**
	AHR	0.104	*		0.087	0.06
Treg	FOXP3	-0.039	0.38		-0.024	0.60
	CD25(IL2RA)	-0.041	0.35		-0.01	0.83
	CCR8	0.033	0.46		0.038	0.41
T cell exhaustion	PD-1 (PDCD1)	0.112	*		0.11	*
	CTLA4	0.018	0.68		0.017	0.71
	LAG3	0.176	***		0.189	***
	TIM-3 (HAVCR2)	0.25	***		0.257	***
Macrophage	CD68	0.245	***		0.25	***
	CD11b (ITGAM)	0.252	***		0.265	***
M1	INOS (NOS2)	0.072	0.10		0.082	0.07
	IRF5	0.276	***		0.288	***
	COX2(PTGS2)	-0.044	0.32		-0.043	0.35
M2	CD163	0.088	*		0.089	0.05
	ARG1	-0.018	0.69		-0.019	0.67
	MRC1	-0.047	0.29		-0.03	0.52
	MS4A4A	0.16	***		0.174	***
TAM	CCL2	0.073	0.10		0.062	0.17
	CD80	0.165	***		0.161	***
	CD86	0.221	***		0.228	***
	CCR5	0.236	***		0.232	***
Monocyte	CD14	0.211	***		0.203	***
	CD16(FCGR3B)	-0.001	9.74-01		0.002	0.97
	CD115 (CSF1R)	0.238	***		0.25	***
Neutrophil	CD66b (CEACAM8)	0.06	0.17		0.054	0.24
	CD15(FUT4)	0.313	***		0.313	***
	CD11b (ITGAM)	0.252	***		0.265	***
Natural killer cell	XCL1	-0.012	0.78		0.003	0.94
	CD7	0.192	***		0.206	***
	KIR3DL1	-0.115	**		-0.116	*
Dendritic cell	CD1C(BDCA-1)	0.042	0.35		0.054	0.24
	CD141(THBD)	-0.005	0.91		0.006	0.90
	CD11c (ITGAX)	0.279	***		0.285	***

Tfh Follicular helper T cell, Th T helper cell, Treg Regulatory T cell, TAM Tumor-associated macrophage. None, correlation without adjustment. Purity, correlation adjusted by purity. Cor, R-value of Spearman's correlation. *P < 0.05; **P < 0.01; ***P < 0.001.

### Pan-cancer analysis of the correlation between the XRCC1 expression and immune checkpoint genes

**Figure 12:**
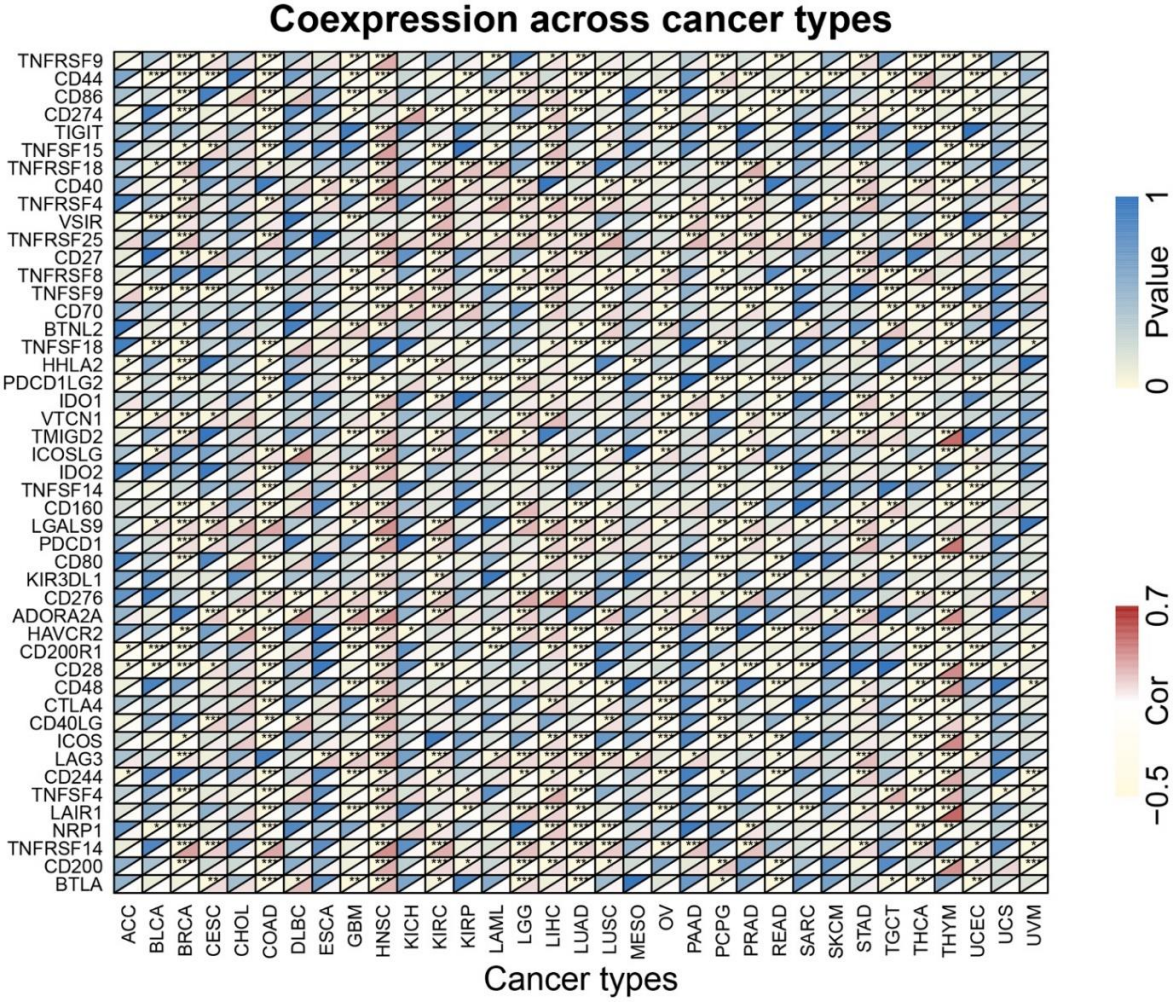
**The association heatmaps between XRCC1 expression and immune checkpoint genes in pan-cancer.** *P < 0.05, **P < 0.01, ***P < 0.001.

The study evaluated the correlation between XRCC1 expression and the expression of immune checkpoint genes, which are crucial in regulating the immune response in various tumors. A total of 47 immune checkpoint genes, including both immunosuppressive and immunostimulatory genes, were analyzed in multiple cancer types (Figure 12). The results revealed a notable correlation between XRCC1 expression and most of the immunosuppressive and immunostimulatory genes in BRCA, COAD, HNSC, LIHC, and THYM. It is worth mentioning that in HNSC, the genes showed a positive correlation with XRCC1 expression, while in COAD, they exhibited a negative correlation. These findings suggest that XRCC1 may have a role in modulating the pattern of tumor immunity by regulating the expression levels of these immune checkpoint genes in specific tumor types.

### Functional enrichment analysis of XRCC1 in LGG

**Figure 13:**
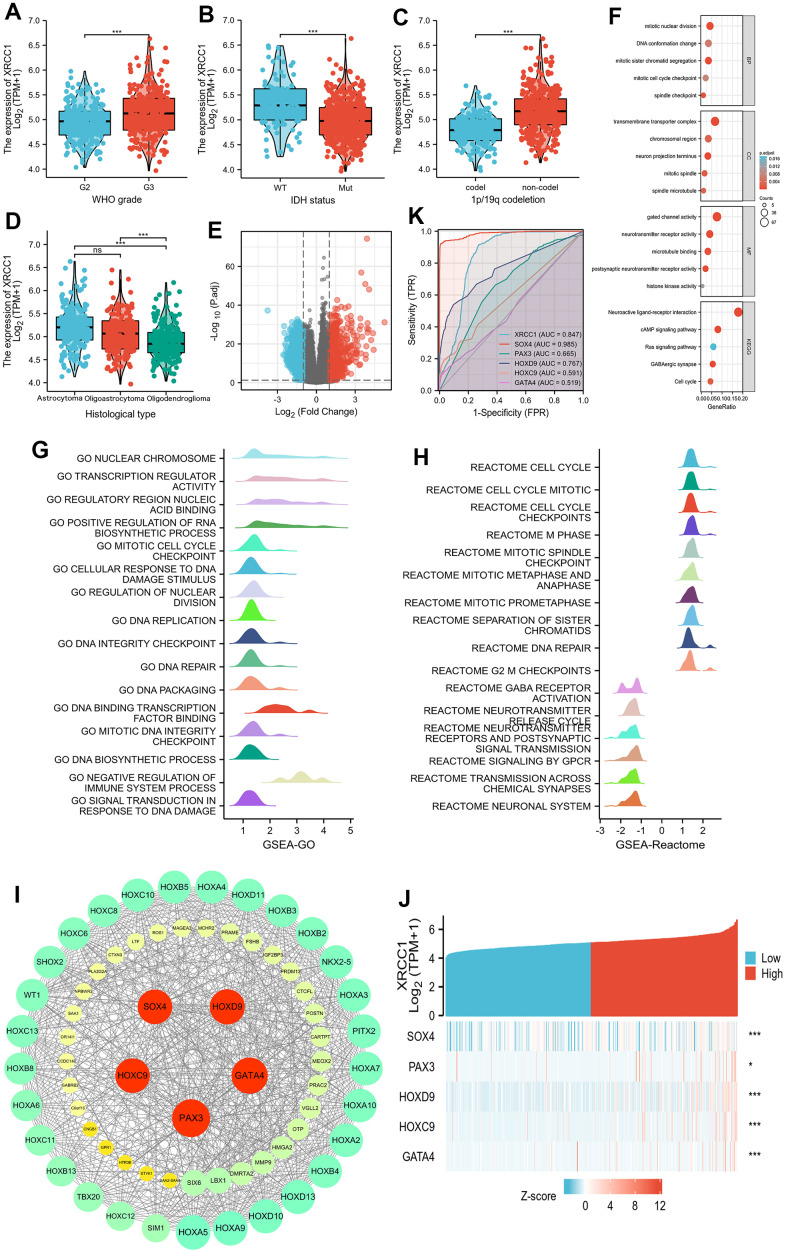
**Associations between XRCC1 expression and different clinical characteristics in LGG.** PPI network building and GO, KEGG, and GSEA analyses between XRCC1 high and low expression groups in LGG. Hub genes positively correlated with XRCC1 expression in LGG and hub genes' receiver operating characteristic (ROC) curve. (**A**) WHO grade. (**B**) IDH status. (**C**) 1p/19q codeletion. (**D**) Histological type (ns, p≥0.05, *p<0.05, **p<0.01, ***p<0.001). (**E**) volcano plot of DEGs (red: upregulation, blue: downregulation). (**F**) GO and KEGG analyses of DEGs. (**G**, **H**) significant GSEA results for DEGs, including GO terms and Reactome pathways. (**I**) PPI network. (**J**) the gene coexpression heatmap of the hub genes. (**K**) ROC curve of hub genes.

In the study of LGG patients, a total of 529 primary LGG cases from the TCGA database were analyzed to investigate the relationship between XRCC1 expression levels and clinicopathological characteristics (Table 2). The patients were divided into low-expression (n=264) and high-expression (n=264) groups based on the mean XRCC1 relative expression. Statistical tests such as chi-square test, Fisher's exact test, and Wilcoxon signed rank sum test were performed to analyze the associations. The results indicated significant associations between XRCC1 expression levels and several clinicopathological characteristics of LGG. XRCC1 expression was found to be correlated with WHO grade (P<0.001), IDH status (P<0.001), 1p/19q codeletion (P<0.001), and histological type (P<0.001) (Figure 13A–13D). To further confirm these findings, logistic regression analysis was conducted, and the results supported the associations between XRCC1 expression levels and the clinicopathological characteristics of LGG (Table 3). Furthermore, gene expression analysis identified a total of 1832 differentially expressed genes (DEGs) in LGG patients, including 677 upregulated genes and 1155 downregulated genes, using predefined thresholds for fold-change and adjusted p-values (Figure 13E). Among these DEGs, 134 genes exhibited significant differential expression, including 94 upregulated genes and 40 downregulated genes. Gene Ontology (GO) and KEGG pathway enrichment analyses were performed to explore the functional implications of these DEGs (Figure 13F and Supplementary Table 1). The GO analysis revealed that the DEGs were enriched in biological processes (BP) such as mitotic sister chromatid segregation, mitotic nuclear division, spindle checkpoint, DNA conformation change, and mitotic cell cycle checkpoint. In terms of cellular component (CC), the enriched categories included transmembrane transporter complex, neuron projection terminus, mitotic spindle, chromosomal region, and spindle microtubule. The molecular function (MF) analysis highlighted neurotransmitter receptor activity, postsynaptic neurotransmitter receptor activity, gated channel activity, microtubule binding, and histone kinase activity. Additionally, the KEGG pathway analysis revealed significant associations with GABAergic synapse, neuroactive ligand-receptor interaction, cAMP signaling pathway, cell cycle, and Ras signaling pathway. These findings provide valuable insights into the molecular characteristics and functional pathways associated with XRCC1 expression in LGG patients, shedding light on its potential role in LGG progression and suggesting potential therapeutic targets.

**Table 2:** Correlation between XRCC1 expression and clinicopathological characteristics in LGG.

**Characteristic**	**Low expression of XRCC1**	**High expression of XRCC1**	**p**
n	264	264	
WHO grade, n (%)			< 0.001
G2	133 (28.5%)	91 (19.5%)	
G3	104 (22.3%)	139 (29.8%)	
IDH status, n (%)			< 0.001
WT	25 (4.8%)	72 (13.7%)	
Mut	238 (45.3%)	190 (36.2%)	
1p/19q codeletion, n (%)			< 0.001
codel	132 (25%)	39 (7.4%)	
non-codel	132 (25%)	225 (42.6%)	
Primary therapy outcome, n (%)			0.073
PD	42 (9.2%)	68 (14.8%)	
SD	76 (16.6%)	70 (15.3%)	
PR	34 (7.4%)	30 (6.6%)	
CR	73 (15.9%)	65 (14.2%)	
Gender, n (%)			0.727
Female	122 (23.1%)	117 (22.2%)	
Male	142 (26.9%)	147 (27.8%)	
Race, n (%)			0.866
Asian	3 (0.6%)	5 (1%)	
Black or African American	11 (2.1%)	11 (2.1%)	
White	242 (46.8%)	245 (47.4%)	
Age, n (%)			0.139
<=40	141 (26.7%)	123 (23.3%)	
>40	123 (23.3%)	141 (26.7%)	
Histological type, n (%)			< 0.001
Astrocytoma	67 (12.7%)	128 (24.2%)	
Oligoastrocytoma	61 (11.6%)	73 (13.8%)	
Oligodendroglioma	136 (25.8%)	63 (11.9%)	
Laterality, n (%)			0.739
Left	126 (24.1%)	130 (24.9%)	
Midline	4 (0.8%)	2 (0.4%)	
Right	131 (25%)	130 (24.9%)	
Age, median (IQR)	40 (32.75, 51)	41.5 (31, 55)	0.484

Abbreviations: CR, complete response; PD, progressive disease; SD, stable disease; PR, partial response.

**Table 3:** XRCC1 expression associated with clinicopathologic characteristics (Logistic Regression).

**Characteristics**	**Total (N)**	**Odds ratio (OR)**	**P-value**
WHO grade (G3 vs. G2)	467	1.953 (1.354-2.829)	<0.001
IDH status (Mut vs. WT)	525	0.277 (0.167-0.448)	<0.001
1p/19q codeletion (non-codel vs. codel)	528	5.769 (3.834-8.840)	<0.001
Primary therapy outcome (PR&CR vs. PD&SD)	458	0.759 (0.524-1.098)	0.144
Gender (Male vs. Female)	528	1.079 (0.766-1.521)	0.662
Race (White vs. Asian&Black or African American)	517	0.886 (0.418-1.858)	0.748
Age (>40 vs. <=40)	528	1.314 (0.934-1.852)	0.117
Histological type (Oligoastrocytoma&Oligodendroglioma vs. Astrocytoma)	528	0.361 (0.249-0.520)	<0.001
Laterality (Right vs. Left&Midline)	523	0.977 (0.693-1.377)	0.896

Gene Set Enrichment Analysis (GSEA) was used to search for GO and Reactome pathways, which revealed that the DNA replication, DNA integrity checkpoint, DNA repair, and signal transduction in response to DNA damage were significantly enriched (Figure 13G and Supplementary Table 2). In addition, Reactome pathway analysis significantly enriched the cell cycle, cell cycle checkpoints, DNA repair, and neuronal system (Figure 13H). These results suggest that XRCC1 is associated with DNA repair-related pathways. Furthermore, we obtained the top 5 hub genes of 134 DEGs, including SOX4, PAX3, HOXD9, HOXC9, and GATA4 (Figure 13I). Moreover, the receiver operating characteristic (ROC) curve was carried out to analyze the effectiveness of hub gene expression in normal samples of GTEx-combined adjacent LGG tissues and LGG samples. XRCC1 (AUC=0.847), SOX4 (AUC=0.985), and HOXD9 (AUC=0.767) indicated that these three genes could be ideal biomarkers to distinguish LGG from non-tumor tissues (Figure 13K). The corresponding heat map data also showed that in LGG, XRCC1 was positively associated with the five genes mentioned above (Figure 13J).

### Analysis of genetic mutation and methylation levels of XRCC1 and hub-gene and pathway regulation in LGG

**Figure 14:**
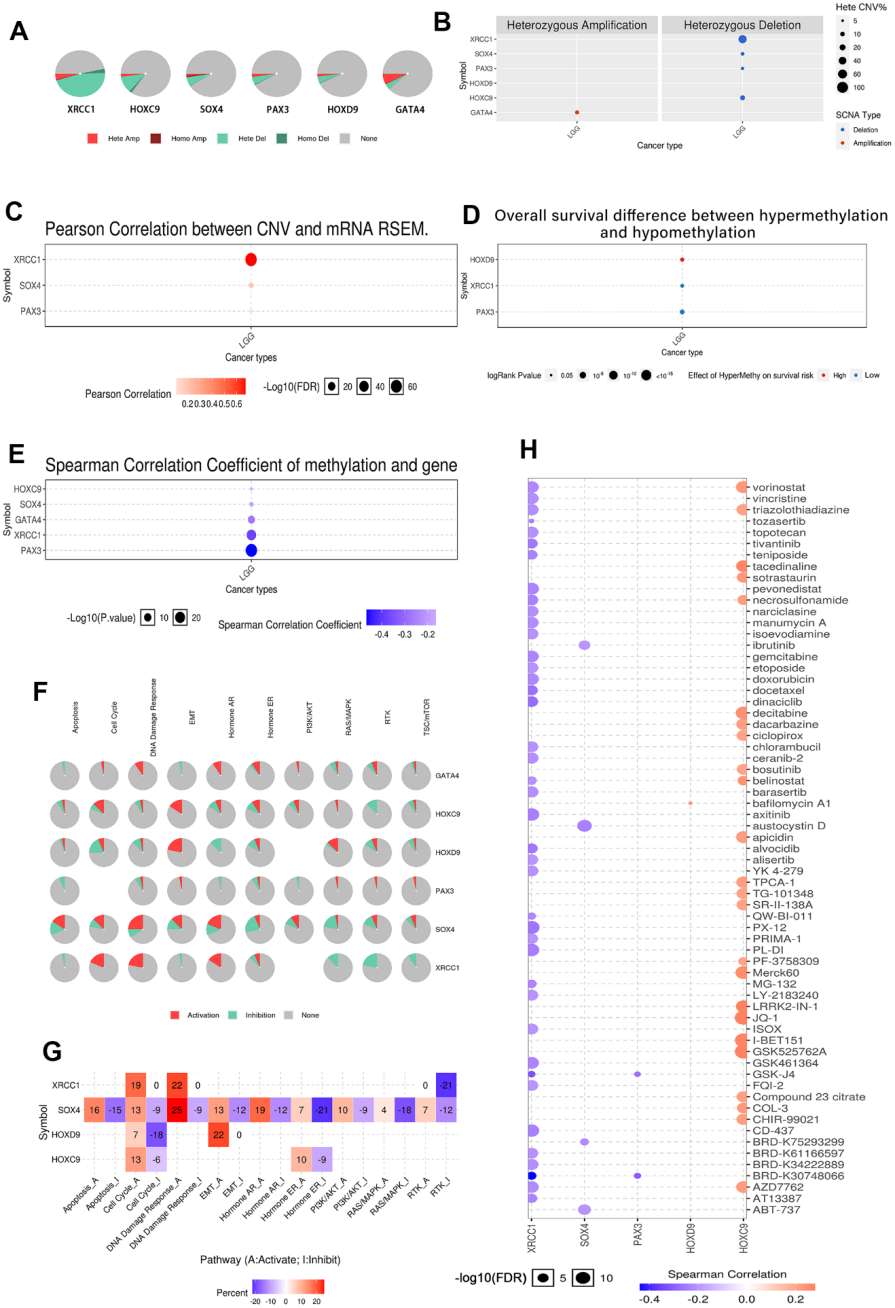
**Analysis of genetic mutation and methylation levels of XRCC1 and hub-gene and pathway regulation in LGG.** (**A**) Homozygous/heterozygous CNV of XRCC1 and its related genes in LGG. Homo Amp: homozygous amplification; Hete Amp: heterozygous amplification; Homo Del: homozygous deletion; Hete Del: heterozygous deletion; None: without CNV. (**B**) The heterozygous CNV of XRCC1 and (**C**) the correlation between CNV and mRNA RSEM. The heterozygous CNV of XRCC1 and the correlation between CNV and mRNA RSEM in LGG were plotted to utilize GSCALite. (**D**) The overall survival discrepancy between hypermethylation and hypomethylation of XRCC1 and its related genes in LGG. (**E**) In the correlation between methylation and gene expression in LGG, blue represented a negative correlation, while red represented a positive correlation. (**F**) XRCC1 and hub genes influence pathways that participate in the development, growth, and progression of LGG. (**G**) The inferred activity of the identified four target genes in pathways that participate in the development, growth, and progression of LGG. A and I to mark the active and inhibited pathways, respectively. (**H**) The correlation of XRCC1 expression and LGG drug sensitivity.

We performed co-analysis of XRCC1 with its correlated genes, namely GATA4, HOXC9, HOXD9, PAX3, and SOX4. The CNV states of these genes in LGG were investigated, as depicted in Figure 14A. XRCC1 exhibited a high susceptibility to mutation, primarily heterozygous deletion, while GATA4 showed heterozygous amplification as the main CNV type. The CNV analysis in Figure 14B confirmed the vulnerability of these genes to heterozygous deletions, with XRCC1 being the most mutation prone. Figure 14C demonstrated a significant correlation between CNV and XRCC1 expression levels in LGG, while a weak correlation was observed for SOX4 and PAX3. Survival analysis based on hypermethylation and hypomethylation patterns (Figure 14D) revealed that hypermethylation of HOXD9 was associated with a higher survival risk in LGG, whereas hypermethylation of XRCC1 and PAX3 indicated a lower survival risk. Furthermore, in Figure 14E, the expression levels of XRCC1 and its correlated genes exhibited negative correlation with methylation levels. Notably, XRCC1 and PAX3 showed substantial statistical significance, suggesting their deregulation. Figure 14F illustrated the influence of these six genes on ten LGG-related pathways. Increased expression of XRCC1 in LGG activated the Cell cycle, DNA damage response, and Hormone AR pathways, while inhibiting Apoptosis, EMT, RAS/MAPK, RTK, and TSC/mTOR pathways. The DNA damage response and EMT pathways were predominantly activated, whereas the RAS/MAPK and RTK pathways were primarily inhibited. Both Apoptosis and PI3K/AKT pathways showed elements of activation and/or inhibition. Moreover, Figure 14H demonstrated the correlation between XRCC1 and its correlated genes' expression levels and drug sensitivity in LGG for specific drugs. Notably, XRCC1 expression was negatively correlated with drug sensitivity in LGG, potentially explaining its classification as a pro-cancer gene. Conversely, HOXC9 expression exhibited a positive correlation with drug sensitivity in LGG. SOX4, PAX3, and HOXD9 displayed limited relevance to drugs.

## DISCUSSION

DNA repair is critical in protecting the cellular genome from damage caused by carcinogens or ionizing radiation [47]. XRCC1 is required for DNA single-strand break repair in human cells. The reduced DNA single-strand break repair and genomic instability caused by XRCC1 defects may contribute to cancer development [48]. A large number of studies have reported that XRCC1 is associated with the development, progression, and prognosis of a variety of cancers, such as thyroid cancer, gastric cancer, non-small cell lung cancer, and cervical cancer. Moreover, XRCC1 Arg194Trp and XRCC1 Arg399Gln were revealed to be strongly associated with susceptibility to glioma among the Chinese population according to a large case-control study [49]. Still, the exact mechanism is unclear [47, 48, 50–55]. Therefore, we systematically analyzed XRCC1 concerning expression, prognostic types, immune invasion, methylation, phosphorylation, and molecular typing in various cancers.

High XRCC1 gene expression was associated with poor OS in LAML and LGG and poor DFS in LGG, LIHC, and LUAD. That is, increased expression of XRCC1 corresponds to a poor prognosis in most cancers. XRCC1, a gene closely related to DNA damage repair, plays an important role in tumor resistance to radiotherapy and chemotherapy. Radiotherapy has been reported to be more effective in patients with non-small cell lung cancer who have XRCC1 gene mutations [56]. This phenomenon also exists in breast, head, neck and rectal cancer [57–59]. In addition, chemotherapy's effect and toxicity were greater in patients with XRCC1 mutated non-small cell lung and gastric cancers than in those with high XRCC1 expression [60–62]. All this evidence suggests that XRCC1 is closely associated with tumor resistance. The main mechanism of action of chemotherapy and radiotherapy is to damage the DNA of tumor cells, and the effectiveness depends on the ability to damage the DNA [63, 64]. Thus, DNA repair interferes with chemotherapy and radiotherapy. For example, glycogen synthase kinase-3β, DNA-dependent protein kinase, and poly (ADP-ribose) polymerase family with DNA repair capabilities reduce the sensitivity of tumors to chemotherapy and radiotherapy [65–67]. At the same time, the enzyme complex formed by XRCC1 is optimized for single-strand break repair and also participates in other repair pathways, playing an important role in DNA repair [68, 69]. Therefore, it is likely that XRCC1 contributes to the poor prognosis of tumor patients by promoting DNA repair and reducing the sensitivity of tumors to chemotherapy and radiotherapy.

DNA methylation and histone modifications such as methylation and phosphorylation are common epigenetic modifications that play an important role in the transcriptional regulation of genes involved in cell cycle progression, proliferation, apoptosis and cell death [70]. Alterations in the epigenetically controlled expression of these genes may also mediate oncogenic processes [71]. It has been demonstrated that XRCC1 phosphorylation by CK2 is required for its stability and efficient DNA repair and that CK2 phosphorylation of XRCC1 facilitates dissociation from DNA and single-strand break formation during BER [72]. In the present study, we found that the T453 site of XRCC1 protein exhibited higher phosphorylation levels in the breast, colon, LUAD, UCEC, and PAAD and that the expression status of XRCC1 phosphorylated proteins (S241, S266, S268, S447, S461 S475, and T453 sites) in certain tumor tissues and normal tissues was somewhat of a difference. This suggests that XRCC1 is also associated with phosphorylation in the development of various types of cancer. In addition, we found a mutation rate of XRCC1 of 1.8% in all tumor patients and a copy number deletion of XRCC1 in all DLBC cases. This suggests that mutations in XRCC1 are associated with cancer development and progression.

Another important finding of this study is that XRCC1-associated infiltration of various immune cells correlates with cancer prognosis. Prognostic analysis showed that high XRCC1 gene expression was associated with poor OS and DFS in LGG. The immune cell infiltration results demonstrated that T cell CD4+ was positively correlated with XRCC1 in LGG. It is well known that T cell CD4+ epitopes are important targets of immunity against infectious diseases and cancer [73]. Several studies have shown that T cell CD4+ is associated with poor prognosis of tumors [74–76], which coincides with the results of our research. Therefore, the high expression of XRCC1 leads to poor prognosis in LGG and is likely to be associated with T cell CD4+ infiltration.

On the other hand, XRCC1 was negatively correlated with B cells (p<0.001). Numerous studies have shown that B cell has a beneficial effect on cancer prognosis [77–80], which supports our view from another perspective. Overall, XRCC1-mediated infiltration of various immune cells in the tumor microenvironment is likely to be closely associated with tumor prognosis. In addition, XRCC1 was significantly associated with most immunosuppressive and immunostimulatory genes in BRCA, COAD, HNSC, LIHC, and THYM. Immune checkpoints are regulators of the immune system that are essential for self-tolerance and prevent the immune system from attacking cells indiscriminately. However, some cancers can protect themselves from attack by tricking immune checkpoints [81]. And the present study found that XRCC1 was significantly correlated with most immunosuppressive and immunostimulatory genes in some tumors, suggesting that the immune escape mechanism of tumors may be associated with XRCC1. TMB is a genetic signature of tumor tissue, defined as the number of non-genetic mutations per million bases in the studied genomic sequence, and its measurement has been achieved by second-generation sequencing [82]. TMB has shown potential as a predictive biomarker with multiple applications, including the association between different reported TMB levels and patient response to immune checkpoint inhibitor therapy in various cancers [83]. The gene expression level of XRCC1 was strongly associated with TMB status across multiple cancer types, which on the other hand, supports the previous conclusions about XRCC1 and immune checkpoints. Taken together, the above results strongly suggest the potential of XRCC1 as an anti-cancer immunotherapy target.

LGGs are a heterogeneous group of tumors that include predominantly glial histology, including astrocytes and/or oligodendrocytes, and tumors with mixed neuron-glial cell morphology. These tumors are considered Grade I and Grade II according to the current WHO classification [84]. Since XRCC1 expression correlates with the prognosis of each type of LGG, we further explored the clinical features, gene function enrichment, mutations, methylation, and drug resistance aspects of XRCC1 in LGG. Based on our analysis of clinical features and gene expression data of 529 primary LGG cases, we found that XRCC1 expression was not only correlated with WHO pathological classification but also negatively correlated with drug sensitivity in LGG, which may also explain why high XRCC1 expression implies poor prognosis in LGG. In addition, we identified XRCC1 (AUC=0.847), SOX4 (AUC=0.985), and HOXD9 (AUC=0.767) as three molecules that could be ideal biomarkers to distinguish LGG from non-tumor tissues. Furthermore, DNA integrity checkpoint, DNA repair, and signal transduction in response to DNA damage were significantly enriched, and these results suggest that XRCC1 pathways are associated with DNA repair in LGG. These results indicate that XRCC1 and DNA repair-related pathways may play a role in the development of LGG.

Our first pan-cancer analysis of XRCC1 revealed a statistical correlation between XRCC1 expression and clinical prognosis, DNA methylation, protein phosphorylation, TMB, MSI, immune cell infiltration, and immune checkpoints. Our results suggest that XRCC1 can be an independent prognostic and diagnostic factor for many tumors. Its expression levels lead to different prognostic outcomes and tumour diagnostic results. However, despite our comprehensive and systematic analysis of XRCC1 and the use of different public databases for cross-validation, this study still has some limitations. First, the gene microarray and sequencing data from different databases exhibited differences and lacked granularity and specificity, which may cause systematic bias. Future studies should rely on higher resolution methods, such as single-cell RNA sequencing, to overcome this problem. Second, *in vivo*/*in vitro* experiments are needed to demonstrate our results regarding the potential function of XRCC1 in LGG, which may increase the credibility of our results. Third, we could not demonstrate that XRCC1 expression affects patient survival through immune infiltration, even though we found that XRCC1 expression was associated with immune cell infiltration in tumors and patient survival. Therefore, further prospective studies are needed to explore the relationship between XRCC1 expression and immune infiltration in cancer patient populations.

## CONCLUSIONS

We conducted a comprehensive investigation into the potential of XRCC1 as a valuable diagnostic and prognostic indicator in diverse cancer types. The positive correlation between XRCC1 expression and immune cell infiltration suggests its involvement in the tumor immune microenvironment. Moreover, the heterogeneity of XRCC1 is highlighted by its differential expression across immunological and molecular subtypes of different malignancies. In certain tumors, XRCC1 may play a role in modulating the tumor immune landscape by regulating the expression of immune checkpoint genes. Overall, XRCC1 emerges as a promising biomarker for cancer diagnosis, prognosis, and immunological assessment, particularly in the context of LGG. This study employs a systems biology approach to analyze the molecular mechanisms behind the development of cancer in an effort to improve our knowledge of cancer biology and yield novel insights for therapeutic approaches. Future investigations ought to concentrate on unraveling the mechanism underlying XRCC1's involvement in various cancer types, particularly in immunomodulation. By closely examining the relationship between XRCC1 and immune cell infiltration, we will seek to elucidate the precise mechanism underlying XRCC1 in regulating tumor immune response. Furthermore, exploring XRCC1's potential role in tumor immunotherapy and assessing its viability as a therapeutic target will provide valuable insights for the design of tailored cancer therapies and immunotherapy approaches.

## Supplementary Material

Supplementary Figures

Supplementary Tables
