# Supplementary material for: XRCC1: a potential prognostic and immunological biomarker in LGG based on systematic pan-cancer analysis
Source: Aging (Albany NY). 2024 Jan 12;16(1):872–910. doi: 10.18632/aging.205426 (PMC10817400; doi:10.18632/aging.205426)
Supplement: Supplementary Figures [file aging-16-205426-s001.pdf]

SUPPLEMENTARY FIGURES

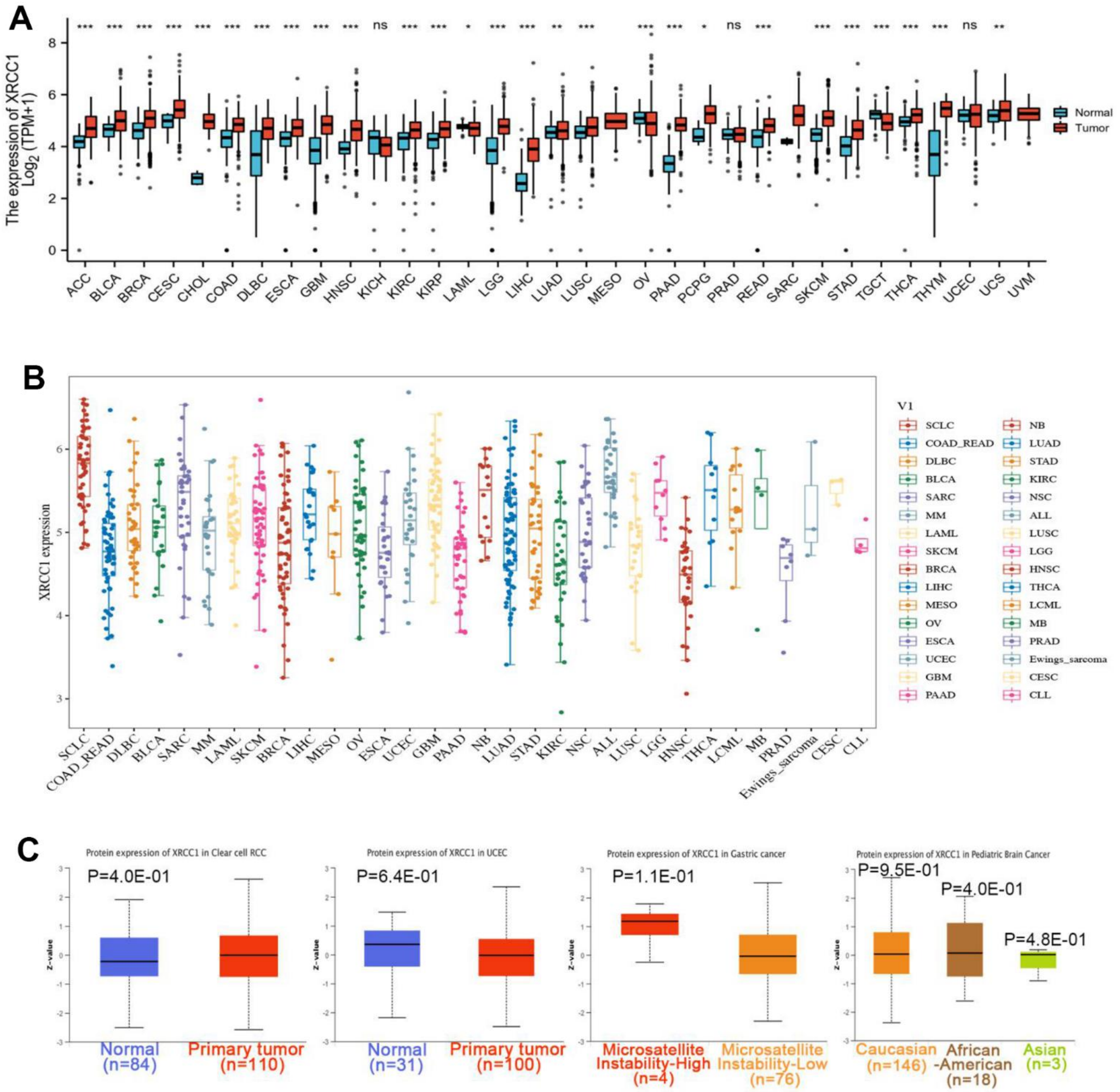

Supplementary Figure 1. Wilcoxon rank sum test was performed to explore the differential expression of XRCC1 between normal and tumor tissues in combination with TCGA and GTEx dataset (**A**). We analyzed the cell line expression matrix of 32 tumors from TCGA database (**B**). The protein expression of XRCC1 genes that were not significantly different was presented (**C**). (ns, P≥0.05; *, P< 0.05; **, P<0.01; ***, P<0.001).

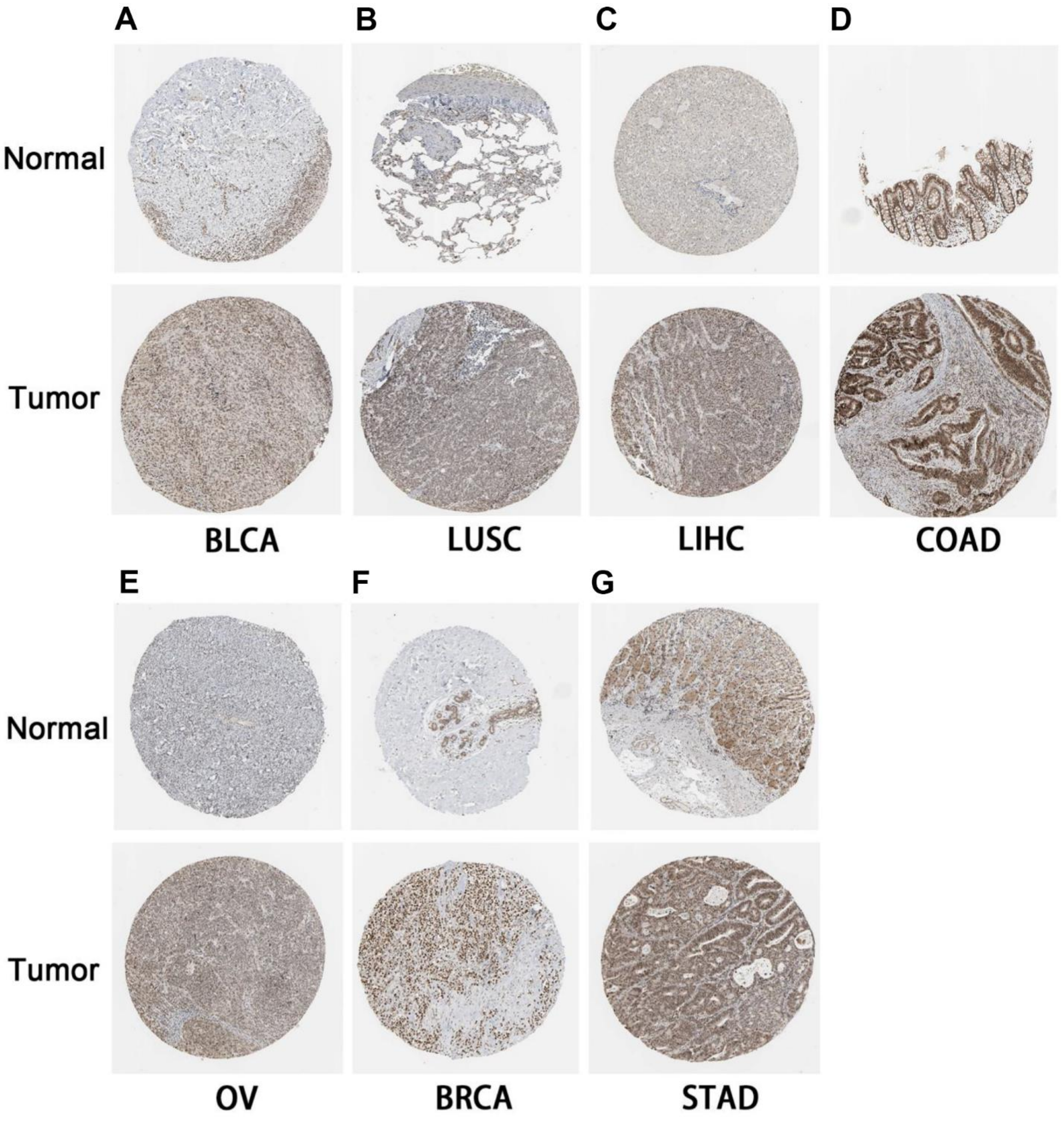

Supplementary Figure 2. HPA database verifies the expression of XRCC1 gene in seven tumors. The expression of XRCC1 gene in BLCA (**A**), LUSC (**B**), LIHC (**C**), COAD (**D**), OV (**E**), BRCA (**F**), and STAD (**G**) is significantly higher than that in the corresponding normal tissues.

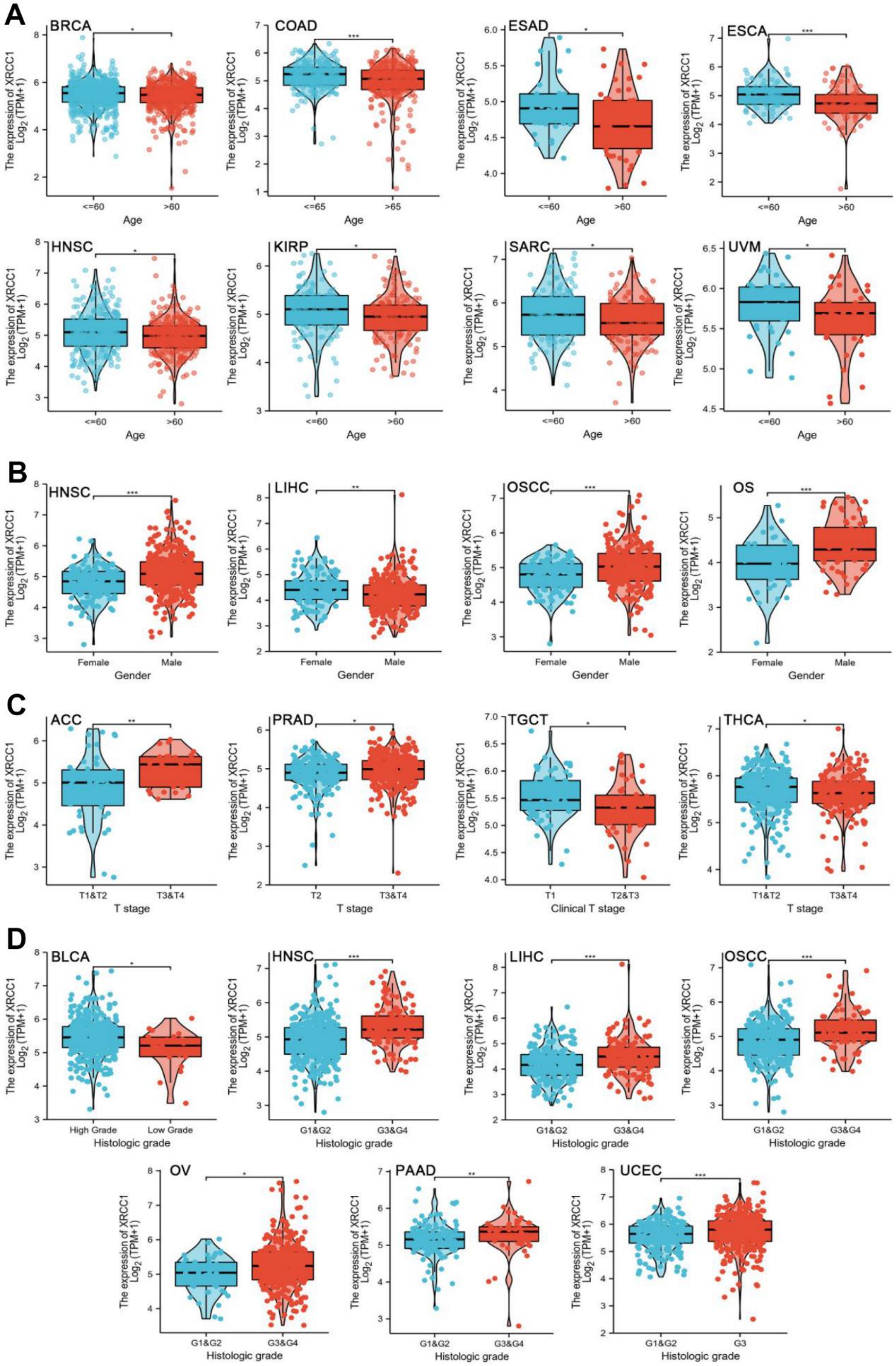

Supplementary Figure 3. Association between XRCC1 gene expression and clinical characteristics, including (**A**) Age, (**B**) Gender, (**C**) T stage, (**D**) Histologic grade.

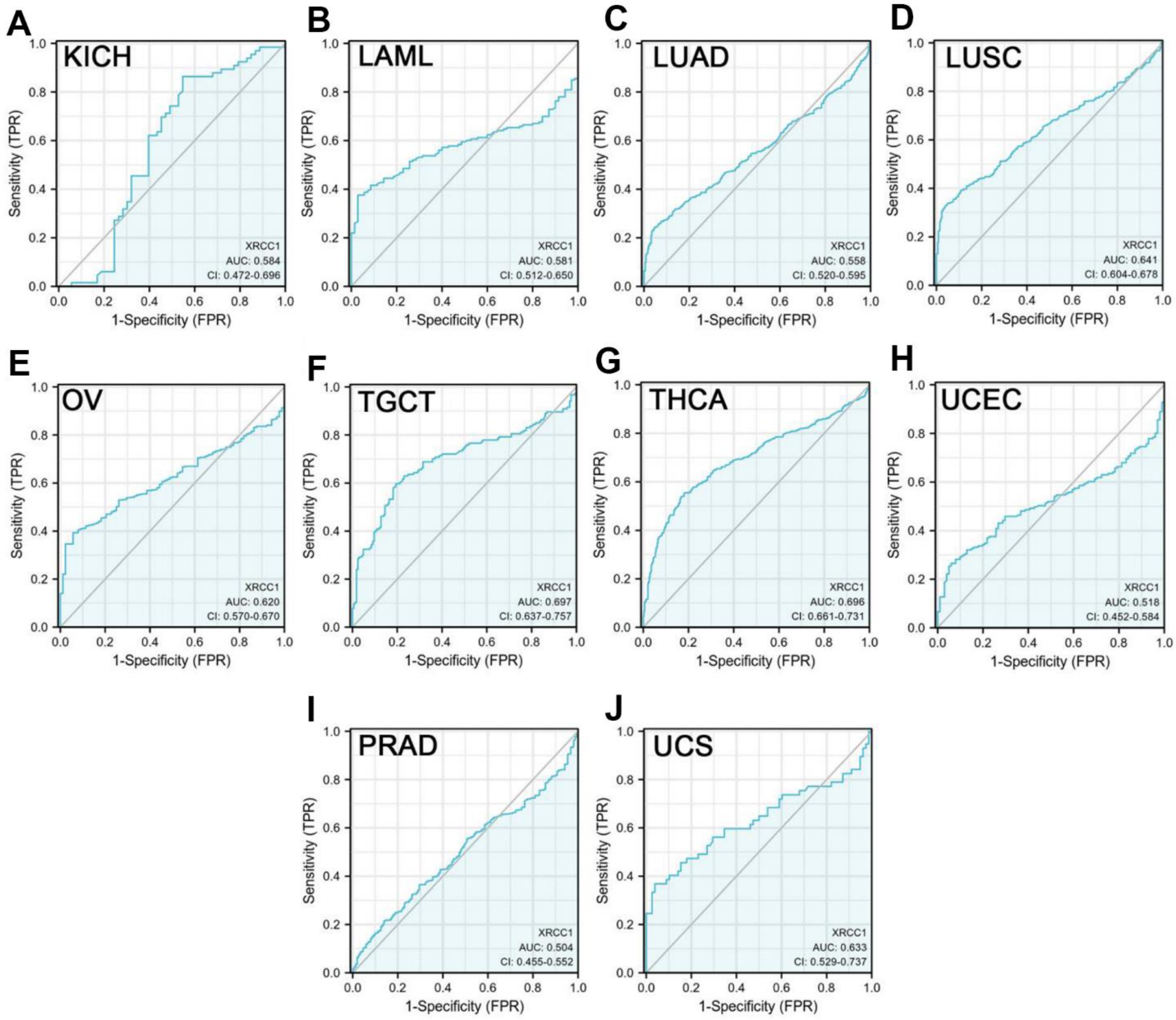

Supplementary Figure 4. ROC curve showed the efficiency of XRCC1 expression level to distinguish tumor tissue from non-tumor tissue. (**A**) KICH, (**B**) LAML, (**C**) LUAD, (**D**) LUSC, (**E**) OV, (**F**) TGCT, (**G**) THCA, (**H**) UCEC, (**I**) PRAD, (**J**) UCS.

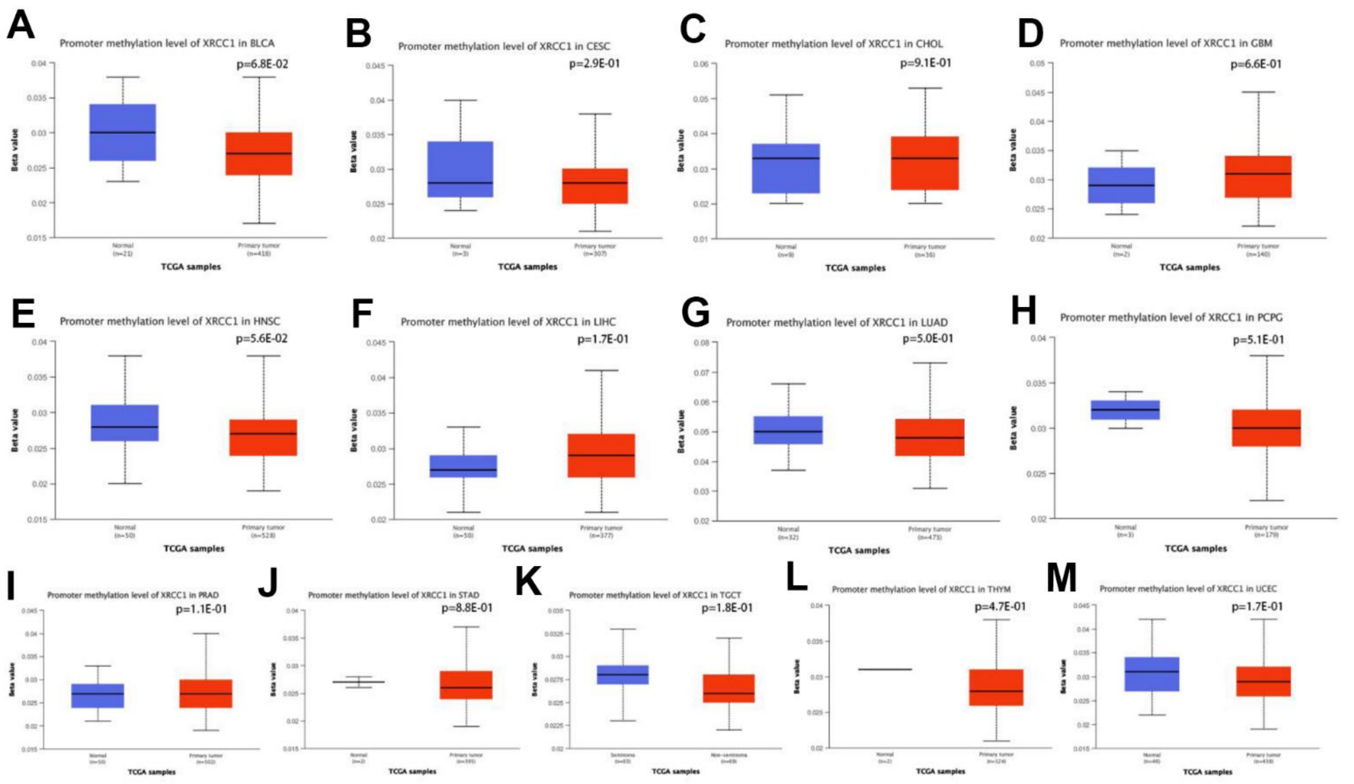

Supplementary Figure 5. Promoter methylation level of XRCC1 in pan-cancer. (**A**) in BLCA, (**B**) in CESC, (**C**) in CHOL, (**D**) in GBM, (**E**) in HNSC, (**F**) in LIHC, (**G**) in LUAD, (**H**) in PCPG, (**I**) in PRAD, (**J**) in STAD, (**K**) TGCT, (**L**) THYM, (**M**) UCEC.

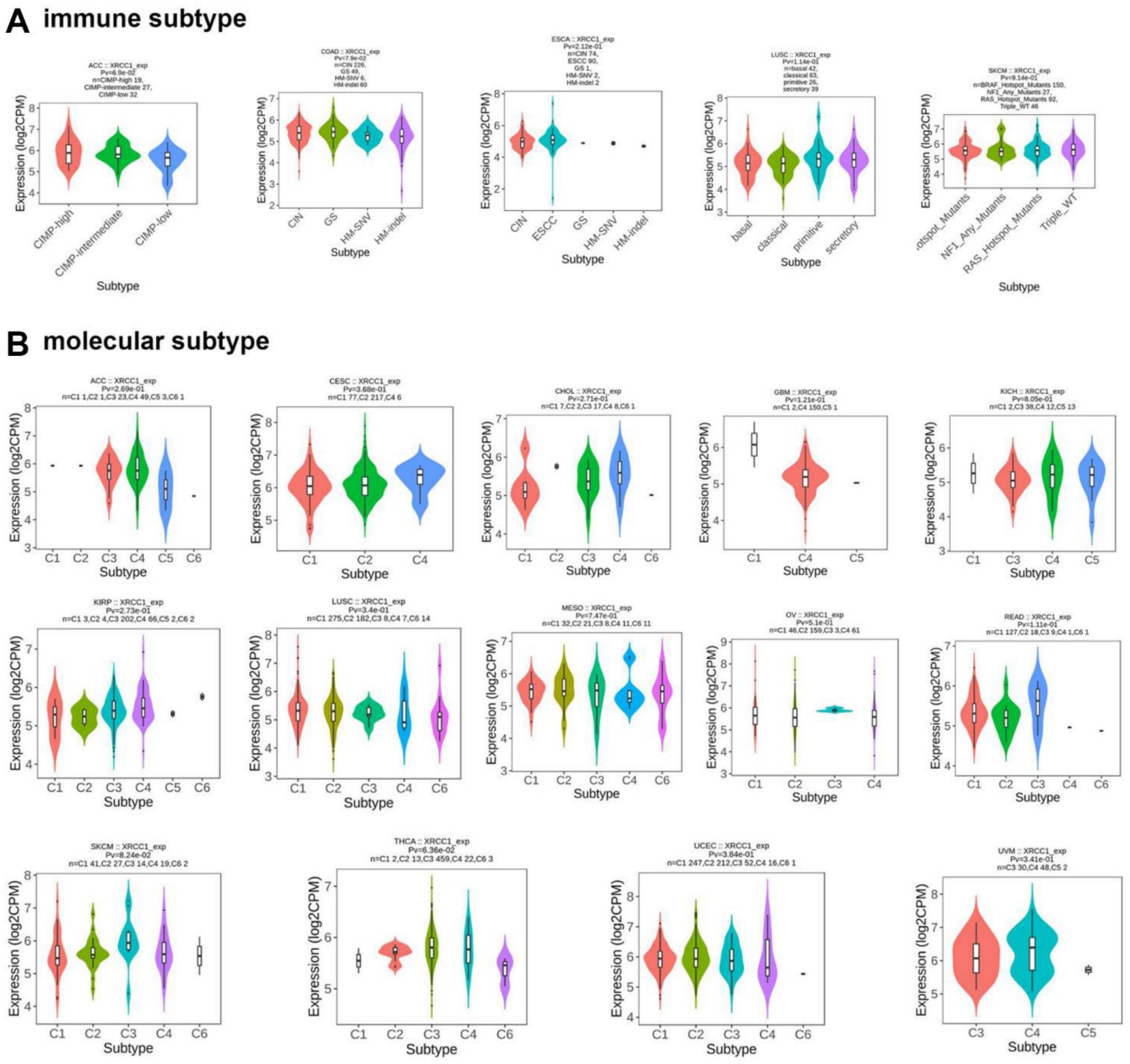

Supplementary Figure 6. The relationship between XRCC1 expression and pan-cancer immune subtypes (**A**) and molecular subtypes (**B**).

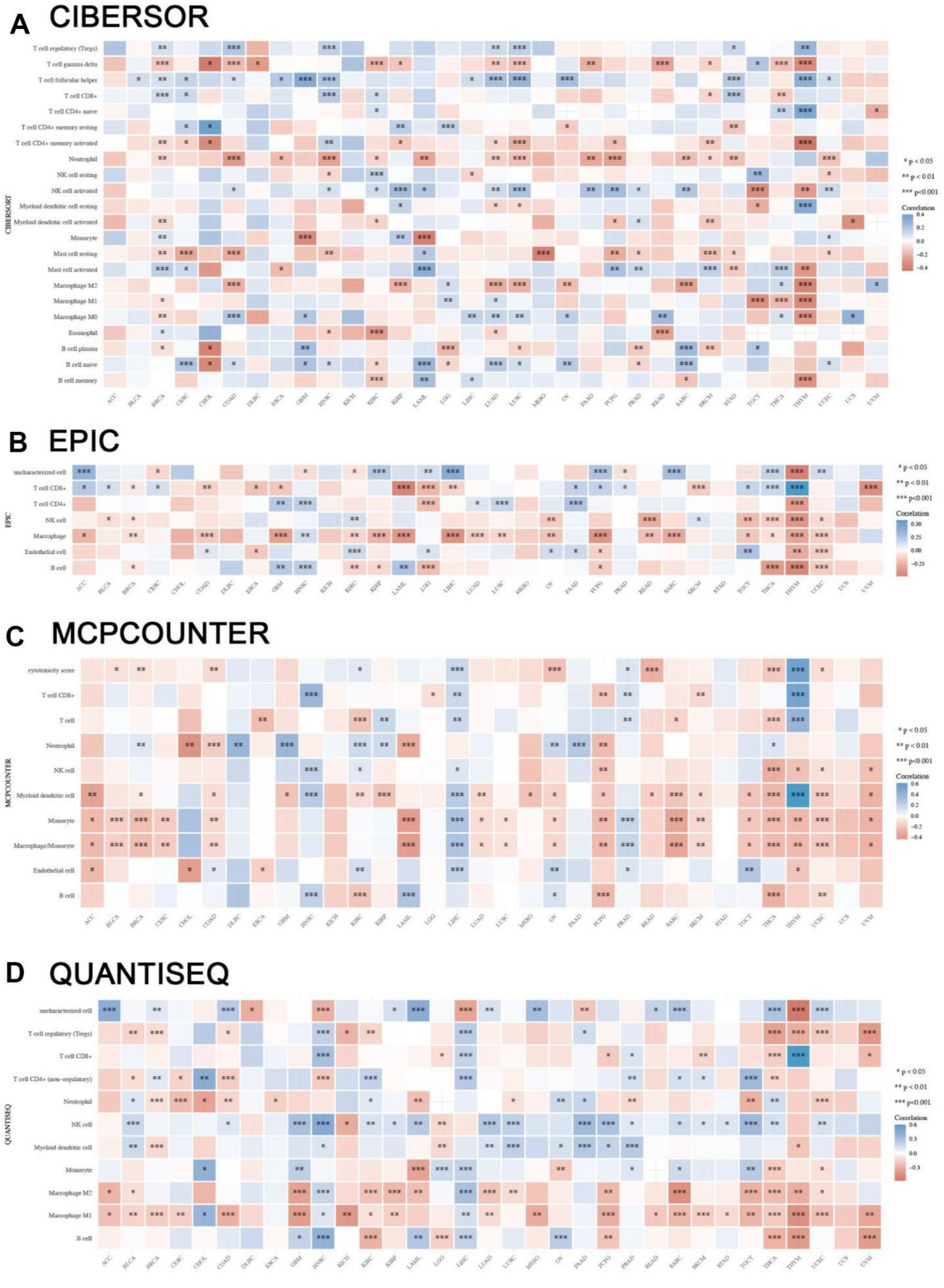

Supplementary Figure 7. We investigated the correlation of XRCC1 gene expression with the level of infiltration of various immune cells via CIBERSOR (**A**), EPIC (**B**), MCPCOUNTER (**C**), QUANTISEQ (**D**). (P≥0.05; *, P< 0.05; **, P<0.01; ***, P<0.001).
