# Supplementary material for: XRCC1: a potential prognostic and immunological biomarker in LGG based on systematic pan-cancer analysis
Source: Aging (Albany NY). 2024 Jan 12;16(1):872–910. doi: 10.18632/aging.205426 (PMC10817400; doi:10.18632/aging.205426)
Supplement: Supplementary Tables [file aging-16-205426-s002.pdf]

SUPPLEMENTARY TABLES

Supplementary Table 1. Results of analyses of GO and KEGG.

Ontology	ID	Description	GeneRatio	BgRatio	P-value	p.adjust	q-value
BP	GO:0000070	mitotic sister chromatid segregation	31/911	151/18670	7.62e-12	1.50e-09	1.29e-09
BP	GO:0140014	mitotic nuclear division	38/911	264/18670	2.13e-09	2.01e-07	1.73e-07
BP	GO:0031577	spindle checkpoint	9/911	34/18670	2.62e-05	7.49e-04	6.47e-04
BP	GO:0071103	DNA conformation change	31/911	327/18670	3.30e-04	0.006	0.005
BP	GO:0007093	mitotic cell cycle checkpoint	19/911	165/18670	4.50e-04	0.008	0.007
CC	GO:1902495	transmembrane transporter complex	63/955	324/19717	1.43e-21	1.74e-19	1.32e-19
CC	GO:0044306	neuron projection terminus	30/955	138/19717	3.00e-12	1.12e-10	8.47e-11
CC	GO:0072686	mitotic spindle	15/955	109/19717	2.43e-04	0.002	0.001
CC	GO:0098687	chromosomal region	32/955	349/19717	4.26e-04	0.003	0.002
CC	GO:0005876	spindle microtubule	10/955	59/19717	4.90e-04	0.003	0.002
MF	GO:0022836	gated channel activity	67/889	343/17697	3.50e-22	1.45e-19	1.22e-19
MF	GO:0030594	neurotransmitter receptor activity	36/889	117/17697	3.24e-19	3.58e-17	3.03e-17
MF	GO:0098960	postsynaptic neurotransmitter receptor activity	18/889	52/17697	2.96e-11	1.35e-09	1.14e-09
MF	GO:0008017	microtubule binding	28/889	246/17697	4.65e-05	7.06e-04	5.98e-04
MF	GO:0035173	histone kinase activity	5/889	17/17697	0.001	0.012	0.010
KEGG	hsa04080	Neuroactive ligand-receptor interaction	65/364	341/8076	1.95e-24	5.19e-22	4.38e-22
KEGG	hsa04727	GABAergic synapse	20/364	89/8076	1.63e-09	8.65e-08	7.29e-08
KEGG	hsa04024	cAMP signaling pathway	29/364	216/8076	1.07e-07	3.56e-06	3.00e-06
KEGG	hsa04110	Cell cycle	16/364	124/8076	1.32e-04	0.002	0.002
KEGG	hsa04014	Ras signaling pathway	21/364	232/8076	0.002	0.017	0.015

Supplementary Table 2. GSEA analysis results.

GSEA-GO

ID	ES	NES
GO_NUCLEAR_CHROMOSOME	0.583	4.832
GO_TRANSCRIPTION_REGULATOR_ACTIVITY	0.586	4.73
GO_REGULATORY_REGION_NUCLEIC_ACID_BINDING	0.558	4.105
GO_POSITIVE_REGULATION_OF_RNA_BIOSYNTHETIC_PROCESS	0.528	4.077
GO_MITOTIC_CELL_CYCLE_CHECKPOINT	0.679	2.983
GO_CELLULAR_RESPONSE_TO_DNA_DAMAGE_STIMULUS	0.541	2.904
GO_REGULATION_OF_NUCLEAR_DIVISION	0.598	2.777
GO_DNA_REPLICATION	0.573	2.619
GO_DNA_INTEGRITY_CHECKPOINT	0.653	2.587
GO_DNA_REPAIR	0.565	2.578
GO_DNA_PACKAGING	0.519	2.475
GO_DNA_BINDING_TRANSCRIPTION_FACTOR_BINDING	0.647	2.433
GO_MITOTIC_DNA_INTEGRITY_CHECKPOINT	0.675	2.379
GO_DNA_BIOSYNTHETIC_PROCESS	0.568	1.924
GO_NEGATIVE_REGULATION_OF_IMMUNE_SYSTEM_PROCESS	0.481	1.906
GO_SIGNAL_TRANSDUCTION_IN_RESPONSE_TO_DNA_DAMAGE	0.549	1.86

GSEA-Reactome

ID	ES	NES	p.adjust
REACTOME_CELL_CYCLE	0.633	4.126	0.006
REACTOME_CELL_CYCLE_MITOTIC	0.632	3.966	0.006
REACTOME_CELL_CYCLE_CHECKPOINTS	0.681	3.769	0.006
REACTOME_M_PHASE	0.587	3.338	0.006
REACTOME_MITOTIC_SPINDLE_CHECKPOINT	0.69	3.22	0.006
REACTOME_MITOTIC_METAPHASE_AND_ANAPHASE	0.626	3.169	0.006
REACTOME_MITOTIC_PROMETAPHASE	0.607	3.127	0.006
REACTOME_SEPARATION_OF_SISTER_CHROMATIDS	0.637	3.101	0.006
REACTOME_DNA_REPAIR	0.658	2.581	0.006
REACTOME_G2_M_CHECKPOINTS	0.676	2.573	0.006
REACTOME_GABA_RECEPTOR_ACTIVATION	-0.484	-1.996	0.012
REACTOME_NEUROTRANSMITTER_RELEASE_CYCLE	-0.58	-2.197	0.007
REACTOME_NEUROTRANSMITTER_RECEPTORS_AND_POSTSYNAPTIC_SIGNAL_TRANSMISSION	-0.394	-2.451	0.007
REACTOME_SIGNALING_BY_GPCR	-0.303	-2.52	0.008
REACTOME_TRANSMISSION_ACROSS_CHEMICAL_SYNAPSES	-0.413	-2.881	0.007
REACTOME_NEURONAL_SYSTEM	-0.414	-3.271	0.008
